# Systematic Review: Does Exercise Training Influence Ghrelin Levels?

**DOI:** 10.3390/ijms26104753

**Published:** 2025-05-15

**Authors:** Wissal Abassi, Nejmeddine Ouerghi, Antonella Muscella, Santo Marsigliante, Moncef Feki, Anissa Bouassida

**Affiliations:** 1 Research Unit “Sport Sciences, Health and Movement” (UR22JS01), High Institute of Sport and Physical Education of Kef, University of Jendouba, Kef 7100, Tunisia; wissalabassi93@gmail.com (W.A.); najm_ouerghi@hotmail.com (N.O.); bouassida_anissa@yahoo.fr (A.B.); 2 Faculty of Medicine of Tunis, University of Tunis El Manar, Rabta Hospital, LR99ES11, Tunis 1007, Tunisia; monssef.feki@gmail.com; 3 High Institute of Sport and Physical Education of Gafsa, University of Gafsa, Gafsa 2100, Tunisia; 4 Department of Biological and Environmental Science and Technologies (DiSTeBA), University of Salento, 73100 Lecce, Italy; santo.marsigliante@unisalento.it

**Keywords:** ghrelin, acute exercise, chronic exercise, growth hormone, muscle metabolism, inflammation

## Abstract

Ghrelin, a gastric-derived peptide, regulates appetite, food intake, and energy homeostasis. Body weight plays a crucial role in modulating circulating ghrelin levels. Since exercise training is one of the most valuable tools for controlling body weight, it is relevant to consider whether exercise can influence total ghrelin secretion. This study aims to perform a systematic review of the effect of acute/chronic exercise on plasma ghrelin levels. An extensive literature search was carried out on various databases, including PubMed, ScienceDirect, and Google Scholar. The search was conducted using English keywords such as acute-exercise, transient-exercise, exercise, chronic-exercise, training, physical-activity, physical-training, exercise training, and total-ghrelin, ghrelin, appetite-related-peptides, gastrointestinal-peptides, gastrointestinal-hormones, and appetite-regulating-hormone. Initially, 2104 studies were identified. After evaluating study quality, data from 61 relevant studies were extracted for inclusion in this review. Most studies indicated that short-term acute aerobic exercise did not affect total ghrelin levels regardless of exercise intensity, characteristics, or growth hormone (GH) secretion. However, long and very-long aerobic/chronic exercise increased total ghrelin levels, mainly in overweight/obese individuals. Acute/chronic exercise may differentially influence total ghrelin secretion. Short-term acute aerobic exercise induces stable plasma ghrelin concentrations, independent of GH secretion. Long-term aerobic training increased its levels mainly in overweight/obese individuals through body composition and oxidative stress reduction. Additionally, total ghrelin secretion is more sensitive to exercise/training duration than exercise/training intensity.

## 1. Introduction

The balance between food intake and energy expenditure is essential to maintaining body homeostasis and health [1]. Appetite control, which involves the sensations of hunger and satiety, is one of the complex physiological processes modulated by peptides released from organs such as the stomach, pancreas, and intestines. These peptides play a critical role in regulating energy balance and nutrient intake [2]. Several gastrointestinal peptides, such as cholecystokinin (CCK), peptide YY (PYY), and glucagon-like peptide 1 (GLP-1), contribute to satiety signaling; however, ghrelin remains the only known circulating factor that stimulates the sensation of hunger and initiates food intake [3].

Following its discovery in 1999 [4], ghrelin was shown to regulate energy homeostasis by establishing an endocrine link between the stomach, hypothalamus, and pituitary via its orexigenic effect, i.e., the ability to stimulate hunger and promote caloric intake, thus linking peripheral signals to central appetite regulation [5].

Apart from its role in appetite regulation, ghrelin affects various physiological processes, including metabolism, energy storage, and muscle function [6]. Ghrelin levels are primarily modulated by the autonomic system, dietary composition, some hormones and metabolic factors, as well as by body weight [7]. Moreover, ghrelin is involved in the release of growth hormone (GH), a peptide that also regulates energy balance and fat metabolism [8]. Both ghrelin and GH are involved in metabolic adaptations to exercise, with GH being known for its role in stimulating muscle growth and fat oxidation [9]. While ghrelin is primarily involved in hunger regulation, GH plays a complementary role in muscle recovery and adaptation to exercise training.

Despite a growing interest in the hormonal responses to physical activity, the current literature on the effects of exercise on ghrelin levels remains inconsistent. While several studies have evaluated both acute and chronic exercise protocols, findings are often contradictory and vary based on factors such as exercise duration, intensity, participant characteristics, and the ghrelin isoform analyzed [10,11,12,13]. Moreover, limited attention has been paid to the interaction between ghrelin and growth hormone in the context of different exercise regimens, despite both hormones playing key roles in energy metabolism, appetite regulation, and body composition [5,8,9,14]. These inconsistencies highlight the need for a comprehensive synthesis of the available evidence to clarify how different types and durations of exercise influence ghrelin regulation. This review seeks to address this gap by systematically examining the effects of exercise, distinguishing between acute and chronic interventions, on circulating ghrelin levels, with particular attention to the role of training duration and its physiological implications.

Physical exercise is widely acknowledged as one of the most significant behavioral factors influencing body weight and, consequently, ghrelin secretion [15,16,17,18,19]. Several studies have examined the variation in ghrelin levels in response to exercise training [9,10,11,12] and have produced conflicting results. Indeed, acute exercise has been shown to either increase [20,21], decrease [11,22], or have no effect on ghrelin levels [23,24]. Similarly, chronic exercise can result in unchanged [25,26], reduced [10,27], or increased [12,28,29] ghrelin levels.

A recent systematic review focusing on ghrelin isoforms provides evidence that acute exercise suppresses acylated ghrelin, whereas chronic exercise leads to an increase in total and deacylated ghrelin [13]. Despite these findings, the exact impact of acute versus chronic exercise on total ghrelin remains uncertain. To clarify these effects, we distinguish between exercise modes and durations. Acute exercise refers to a single session, while chronic exercise refers to structured training interventions conducted over several weeks or months. Additionally, we categorize the duration of a single exercise session as short-term (<60 min), long-term (60–89 min), or very long (≥90 min), and classify chronic exercise intervention programs as short-term (<12 weeks) or long-term (≥12 weeks). Given the importance of exercise in modulating ghrelin, this systematic review aims to provide an updated understanding of how both acute and chronic exercise affect circulating ghrelin levels.

We will first discuss the physiological effects of ghrelin, followed by the impact of short- and long-term acute exercise, and finally, the effects of short- and long-term chronic exercise on ghrelin levels.

Understanding these mechanisms could have implications for developing targeted interventions to manage energy balance, appetite, and metabolic health in both clinical and athletic populations.

### 1.1. Ghrelin

**Figure 1:**
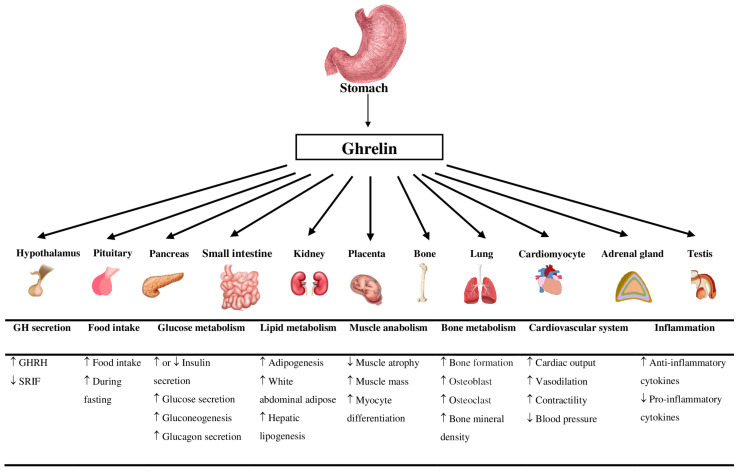
The stomach is the primary source of circulating ghrelin. Ghrelin exerts endocrine effects on multiple target organs, including the hypothalamus, pituitary gland, pancreas, gastrointestinal tract, adipose tissue, and cardiovascular system. Upward arrows (↑) indicate stimulation, while downward arrows (↓) indicate inhibition. Abbreviations: GH, growth hormone; GHRH, growth hormone-releasing hormone; SRIF, somatotropin release-inhibiting factor.

Ghrelin is a 28-amino acid peptide-derived orexigenic hormone [4]. Approximately 60–70% of circulating ghrelin is expressed in the stomach, with most of the remainder originating in the small intestine. Small amounts can also be found in many other human tissues outside the gut, including the pancreas, hypothalamus, pituitary gland, cardiomyocytes, placenta, adrenal gland, lung, kidney, bone, and testes [30,31]. The hormone’s name is based on “ghre”, a root word in Proto-Indo-European languages, which refers to “grow”, regarding its role in the release of growth hormone [4]. Ghrelin is initially formed from preproghrelin, a 117-amino acid peptide, which is then proteolyzed and post-translationally modified, leading to the nascent ghrelin isoforms, acyl-ghrelin (AG) and des-acylghrelin (DAG) [32]. In circulation, approximately 10% of ghrelin is acylated, which refers to its active form, responsible for ghrelin’s major functions. DAG, the inactive form, is the most abundant ghrelin-related molecule in the body, accounting for 80–90% of total circulating ghrelin, exerting primarily non-endocrine functions, including cardioprotective effects [33,34]. The sum of these two forms represents the total ghrelin [33]. Only the acylated form of ghrelin is biologically active at physiological concentrations and capable of binding to and activating the growth hormone secretagogue receptor (GHSR), transferring the information from the stomach to the hypothalamus and influencing GH release in response to changes in energy homeostasis [31]. DAG does not bind GHSR but may have independent biological effects through other mechanisms. Preclinical and clinical data provide evidence for ghrelin as a relevant regulator of nutrient sensing, meal initiation, and appetite [35]. Apart from its orexigenic effect, research over the last decade has identified important effects of ghrelin, such as stimulation of GH secretion, regulation of glucose and lipid metabolism, protection against muscle atrophy, participation in bone formation, modulation of the cardiovascular system, and reduction of inflammation (Figure 1).

### 1.2. Ghrelin and Growth Hormone

**Figure 2:**
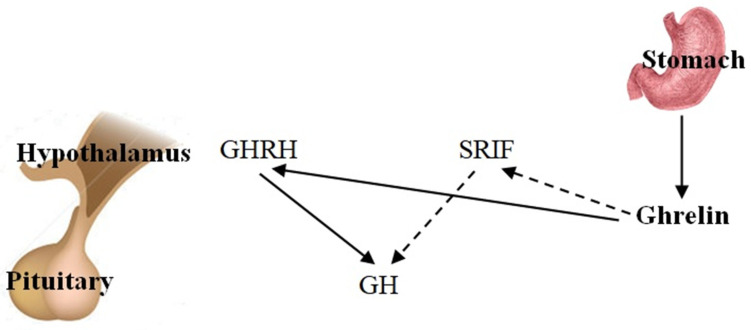
Schematic representation of the regulation of growth hormone (GH) secretion. Ghrelin and GHRH stimulate GH release directly at the pituitary level and via the hypothalamus, while somatostatin (SRIF) inhibits this process. Solid arrows indicate stimulatory actions, and dashed arrows indicate inhibitory actions. Abbreviations: GH, growth hormone; GHRH, growth hormone-releasing hormone; SRIF, somatotropin release-inhibiting factor.

GH is produced and secreted by the somatotrope cells of the anterior pituitary, although it is accepted that other extra pituitary sites produce GH with autocrine or paracrine actions [8]. At the hypothalamic level, GH secretion is controlled dually by two peptides with opposing roles, GH-releasing hormone (GHRH) and somatotropin release-inhibiting factor (SRIF) [36,37]. The GH-releasing action of ghrelin takes place both directly on pituitary cells and through modulation of GHRH from the hypothalamus; some functional anti-somatostatin action has also been shown [8,14,38,39] (Figure 2).

### 1.3. Ghrelin and Food Intake

Ghrelin is well established to stimulate food consumption in both lean and obese humans [40]. The circulating levels of ghrelin are elevated during fasting and before meals [41,42] and decline post-prandially [42,43], which implies that ghrelin plays a significant role in initiating food intake [44]. The increased level of ghrelin during fasting [45] is a unique phenomenon in human physiology, which contrasts with the secretion levels of most gut hormones that increase during nutrient intake and decrease during fasting [44].

### 1.4. Ghrelin and Glucose Metabolism

Numerous studies have shown that ghrelin significantly affects glucose metabolism, primarily by inhibiting insulin secretion, an effect observed across multiple species [46,47]. In the pancreas, ghrelin is produced by both ε-cells and α-cells [46,48,49,50,51]. Although the presence of ε-cells in adult islets remains debated [49], their expansion in animal models is associated with hyperphagia, adiposity, and reduced insulin response to glucose [52]. Ghrelin and its receptor regulate glucose metabolism by inhibiting β-cell insulin release and stimulating α-cell glucagon secretion [53]. Ghrelin O-AcylTransferase inhibition improves glycemic control and enhances insulin secretion [54]. This insulinostatic action involves growth hormone secretagogue receptor type 1a (GHSR1a)/G protein alpha inhibitory subunit (Gαi) signaling and interaction with the somatostatin receptor subtype 5 (SST5) in pancreatic β-cells [55]. Interestingly, ghrelin may also protect β-cells under type 1 diabetes-like conditions, despite its reduced levels in such states [56,57].

### 1.5. Ghrelin and Lipid Metabolism

Ghrelin influences lipid metabolism through both central and peripheral mechanisms. Centrally, ghrelin acts on the hypothalamus to modulate the melanocortin system, leading to the stimulation of lipogenic pathways and a concurrent reduction in fat oxidation [58,59]. Peripherally, ghrelin increases triglyceride accumulation, particularly in the liver and white adipose tissue, suggesting a direct lipogenic role [60,61,62]. Moreover, ghrelin suppresses lipolysis by downregulating hormone-sensitive lipase activity in adipocytes, thereby reducing fatty acid mobilization and oxidation [63,64]. In addition, ghrelin has been reported to enhance adipogenesis and elevate triglyceride content, contributing to increased fat storage in white adipose tissue [61,65]. Specifically, ghrelin promotes fat storage in selective abdominal depots such as retroperitoneal and inguinal fat by reducing lipid export, rather than directly inhibiting lipolysis [66]. Animal studies have shown that ghrelin activates its receptor on hepatocytes to promote lipogenesis [67,68].

These effects may reflect a biological mechanism aimed at conserving energy during periods of caloric restriction or negative energy balance, which is relevant in understanding ghrelin’s physiological role during fasting and exercise, when the hormone is elevated and may serve to protect energy stores [13,69]. However, these same mechanisms could contribute to metabolic dysregulation under chronic ghrelin elevation, such as in obesity or Prader–Willi syndrome, leading to increased fat storage and impaired lipid utilization [31,70].

### 1.6. Ghrelin and Muscle Metabolism

Muscle atrophy associated with aging or various diseases is an important contributor to the reduction of skeletal muscle mass and strength, leading to muscle dysfunction and weakness [6]. The anti-atrophic effects of ghrelin in muscle have been proven [6]. A previous study reported the protective effect of ghrelin in fasting- and denervation-induced muscle atrophy in mice [71]. Interestingly, the anabolic effects of ghrelin are also confirmed in animal studies with various diseases [72,73] by inhibiting skeletal muscle protein degradation. It has been shown that ghrelin can indirectly increase muscle mass by stimulating food intake, activating the GH/Insulin-like growth factor-1 (IGF-1) axis in cachexia mice [74], and promoting myocyte differentiation and fusion in C2C12 myoblasts [75].

### 1.7. Ghrelin and Bone Metabolism

Over the last decade, ample studies have demonstrated the effect of ghrelin on osteoblast function. The majority of the in vitro and in vivo studies reported that ghrelin directly promotes osteoblast proliferation and differentiation in vitro, increases bone mineral density, and reduces apoptosis across different species [76,77,78]. It is also proven that in vivo, ghrelin increases the transcription of genes encoding the osteoblast differentiation markers (collagen type I and osteocalcin), alkaline phosphatase activity, and calcified accumulation [76]. The role of ghrelin in osteoclast biology is also confirmed, as Costa et al. [79] showed that ghrelin enhanced osteoclast activity, differentiation, and resorption in rats. Ghrelin is classified as a GH secretagogue, meaning it stimulates the release of GH by binding to specific receptors in the pituitary gland. Through this mechanism, it has been shown to accelerate skeletal growth [80], increase bone mineral content [81], and enhance bone mineral density in animal studies [76], suggesting a role in bone formation via activation of the GH–IGF-1 axis [82]. Despite the promising effects of exogenous ghrelin on bone metabolism demonstrated in several in vitro and animal studies [76,79,83,84,85,86], evidence from human studies remains limited and inconclusive [87,88,89].

### 1.8. Ghrelin and the Cardiovascular System

Ghrelin participates in regulating various aspects of the cardiovascular system in both physiological and pathophysiological states [4]. It is involved in cardiac performance improvement and blood vessel protection [90]. In healthy subjects, intravenous or subcutaneous ghrelin administration increases cardiac output, improves cardiac contractility, and decreases mean arterial pressure without altering heart rate [91]. At the same time, in patients with chronic heart failure, acute ghrelin administration increases cardiac output, cardiac index, and stroke volume index [92], which can be explained by the decrease in left ventricular afterload under the vasodilatory effect of ghrelin and its central effect on the nucleus of the solitary tract [93]. Ghrelin also regulates the heart’s electrical activity by the suppression of sympathetic nerve activity and activation of parasympathetic nerve activity [94]. It has been shown to preserve cardiac function by reducing cardiomyocyte apoptosis, fibrosis, and cardiac injury [95]. Ghrelin has been shown to decrease blood pressure in healthy humans through its potential vasodilatory effect [91], by nitric oxide-independent mechanisms, via inhibition of the sympathetic nervous system, resulting in low blood levels of norepinephrine and contributing to the vasodilatory effect of ghrelin [96]. Different studies conducted among human subjects have confirmed that ghrelin participates in atherosclerotic plaque stability by reducing vascular endothelial cell apoptosis and vascular inflammation, leading to endothelial function improvement [97,98].

### 1.9. Ghrelin and Inflammation

The beneficial role of ghrelin in upregulating the expression of anti-inflammatory cytokines has been reported in several studies [99,100]. Pre-clinical studies have shown that ghrelin administration enhances interleukin-10 release by stimulating the phosphorylation of the mitogen-activated protein kinase (MAPK) via the growth hormone secretagogue receptor. This mechanism, observed in cell and animal models, contributes to attenuating inflammation by modulating pro-inflammatory cytokines [99,100]. Although a few clinical studies have reported similar anti-inflammatory trends in humans [101,102], further research is needed to confirm these effects in larger and more diverse populations.

Moreover, ghrelin has been shown to suppress inflammation, regulate apoptosis, and reduce damage to different target organs, including the nervous, respiratory, cardiovascular, and gastrointestinal systems, mainly by attenuating the release of pro-inflammatory cytokines [103]. Several in vitro and in vivo results confirm that exogenous ghrelin prevents pro-inflammatory responses in the central nervous system by inhibiting the release of pro-inflammatory cytokines, including interleukin-6, tumor necrosis factor-TNF-α, and interleukin-1β in kainic acid-induced neuronal injury [104]. Some findings indicate the adverse effects of ghrelin on intestinal inflammation. Ghrelin administration to mice with intestinal injury reduced neutrophil infiltration, pro-inflammatory markers, reduced inflammation, and improved organ damage through activation of the protein kinase mTOR signaling pathway [105]. In addition, when ghrelin was administered to the rat model of isoproterenol-induced myocardial infarction, cardiac enzyme levels decreased, electrocardiograms changed, oxidative stress injury was attenuated, and serum and cardiac tissue inflammatory marker levels decreased, including nuclear factor kappa-B, inducible nitric oxide synthase, and interleukin-6 [106].

## 2. Methods

The current review was conducted and reported by the Preferred Reporting Items for Systematic Reviews and Meta-Analysis (PRISMA) guidelines and recommendations [107].

### 2.1. Literature Search Strategy and Study Selection

A systematic search strategy consisting of keywords related to exercise interventions and ghrelin hormone was performed to find relevant articles using PubMed, ScienceDirect, and Google Scholar databases. The search was conducted from database inception up to December 2024. Using the operators “AND”, “OR”, the following essential phrases were added and combined: (“acute exercise” OR “transient exercise” OR “exercise” OR “chronic exercise” OR “training” OR “physical activity” OR “physical training” OR “regular exercise” OR “exercise training”) and (“total ghrelin” OR “ghrelin” OR “appetite-related peptides” OR “gastrointestinal peptides” OR “gastrointestinal hormones” OR “Appetite-Regulating Hormone”). The search strategy was supplemented with hand searches of the reference lists of included full-text articles, as well as through searching related articles and citations in the PubMed database to identify further relevant research papers.

### 2.2. Eligibility Criteria

To be eligible for inclusion in the present study, the screened articles had to meet the following requirements: (a) Publication: written in English and published in a peer-reviewed journal; (b) Participants: individuals regardless of their gender, age, weight, health status and fitness level; (c) Intervention: studies involving either acute exercise (defined as a single exercise bout) or chronic exercise (defined as a multi-week structured training program), regardless of modality or intensity. Exercise duration was further categorized based on two criteria. (d) Comparison: at the minimum, two assessments of ghrelin, pre-and post-exercise or training intervention; (e) Outcome: measurement of ghrelin or total ghrelin levels. Exclusion criteria were as follows: (a) measures of acylated/deacylated, and/or active ghrelin concentrations without ghrelin or total ghrelin measurement; (b) nonrandomized controlled trials (e.g., reviews, conference articles, case reports, cohort study, commentary, guideline); (c) missing or insufficient training or ghrelin data; and (d) animal model publications.

For the purposes of this review, “acute exercise” refers to the effects of a single exercise session, typically lasting from a few minutes to several hours. In contrast, “chronic exercise” refers to repeated exercise sessions performed over an extended period, ranging from several days to several weeks or months.

With regard to the classification of duration, “short-term duration” refers to single exercise sessions measured in minutes or hours. “Short-term intervention” refers to exercise programs lasting up to 4 weeks, while “long-term” interventions range from 4 to 12 weeks, and “very long-term” interventions exceed 12 weeks.

Exercise duration was further categorized based on two criteria. First, the duration of a single exercise session was classified as short-term when it lasted less than 60 min, long-term when it lasted between 60 and 89 min, and very long-term when it lasted 90 min or more. Second, the total duration of the training intervention was considered short-term for programs lasting less than 12 weeks and long-term for those lasting 12 weeks or more.

### 2.3. Data Extraction

After removing all duplicates, the first author screened all titles and abstracts to identify potentially eligible studies based on the inclusion and exclusion criteria. The following data points were extracted from each included study: first author’s last name, year of publication, sample size (n), participant characteristics (age and sex, body mass index (BMI) or body mass and health status), intervention features (modality, type, frequency, intensity, session, and intervention duration), and ghrelin outcome measures (pre/post-intervention mean and direction of change). Data about changes in body mass/fat or BMI and circulating GH were also obtained when available.

### 2.4. Quality Assessment

The methodological quality and risk of bias of the included randomized controlled trials were assessed using the Cochrane Risk of Bias Tool version 2 (RoB 2), developed by the Cochrane Collaboration. This tool evaluates five domains of potential bias, including (1) bias arising from the randomization process, (2) bias due to deviations from intended interventions, (3) bias due to missing outcome data, (4) bias in measurement of the outcome, and (5) bias in selection of the reported result.

Two authors independently assessed each study across these domains and judged the overall risk of bias as “low”, “some concerns”, or “high risk”. Any discrepancies between reviewers were resolved through discussion or, when necessary, by consulting a third reviewer. The results of the quality assessment are summarized in Supplementary Table S1.

## 3. Results

### 3.1. Study Selection Process

**Figure 3:**
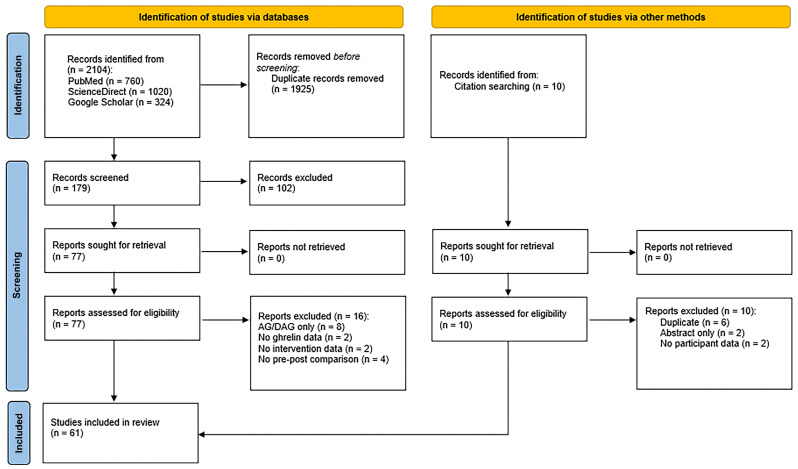
PRISMA 2020 flow diagram for the systematic review, including the identification process, screening, and final number of studies included. AG, acyl-ghrelin, DAG, des-acylghrelin.

The initial electronic database searches led to a total of 2104 records. Ten further studies were found from hand searching of reference lists of included studies. After removing duplications, a wide range of titles, abstracts, and full texts were screened, and a careful assessment was carried out on 61 relevant related studies [10,11,12,19,20,21,22,23,24,25,26,27,28,29,108,109,110,111,112,113,114,115,116,117,118,119,120,121,122,123,124,125,126,127,128,129,130,131,132,133,134,135,136,137,138,139,140,141,142,143,144,145,146,147,148,149,150,151,152,153,154] (Figure 3).

A summary of the included studies’ characteristics and outcomes by exercise mode and duration is displayed in Table 1 and Table 2.

### 3.2. Participant Characteristics

The participant characteristics varied concerning sex, age class, body composition, health status, or training level. Most studies (n = 28) were conducted in males, while 11 studies concerned females, and 20 studies included both sexes. Two studies did not identify the gender of participants [10,28]. Participants were youth/young adults in 41 studies, elderly individuals in 14 studies, and children in 5 studies. One study did not report the participants’ age [10]. The majority of studies (n = 29) involved normal-weight people, while 28 studies investigated overweight/obese people, 3 studies were conducted in both groups, and 1 study concerned lean and normal-weight individuals. Regarding health status, five studies involved clinical populations, including patients with GH deficiency [108], metabolic syndrome [29], osteoarthritis [26], type 2 diabetes mellitus [138,148], and children with precocious puberty [12]. Participants were inactive in 42 studies, moderately active in 5 studies, and athletes in 14 studies. Sample sizes range from 5 to 399 participants, with most studies (70.49%) having fewer than 30 participants.

### 3.3. Exercise Characteristics

Ghrelin responses to acute exercise were assessed in 30 of the 61 included studies, responses to chronic exercise were studied in 30 studies, and response to both acute and chronic exercise was assessed in 1 study [128]. There was considerable heterogeneity across trials concerning exercise/training type, intensity, frequency, and duration. Studies assessing the effects of acute exercise used either aerobic (n = 24), resistance (n = 4), combined (n = 2), or intermittent (n = 1) exercise. Concerning exercise duration, 18, 8, and 4 studies implemented short (<60 min), long (≥60 min), and very long-term (≥90 min) exercise, respectively. One study used both long (≥60 min) and very long-term (≥90 min) exercise [125].

Chronic exercise studies implemented either aerobic (n = 19), resistance (n = 2), combined (n = 8), or intermittent (n = 2) training programs. Most of these studies (n = 19) employed a long-term program (≥12 weeks), while 8 and 2 studies applied a short-term (<12 weeks) or very long-term (≥ 48 weeks) training program, respectively. Two studies used both short (<12 weeks) and long-term (≥12 weeks) [29], or both long (≥12 weeks) and very long-term (≥48 weeks) [143]. The majority of acute or chronic exercise studies used moderate-intensity exercise. Some studies paired acute/chronic exercise with other interventions, including diet [10,21,28,112,131,141,142,144,145,146,147,154], bioenterics intragastric balloon [146], medication [12], GH administration [108] or carbohydrate supplementation [129].

### 3.4. Analytical Characteristics

In the 61 included studies, 33 studies reported “total ghrelin”; however, only 28 studies measured “ghrelin”. High-quality immunoassay methods with optimal precision were used in all studies to assess “total ghrelin/ghrelin” levels, which were measured with different units including (pg. mL^−1^), (fmol·mL−^1^), (pmol. L^−1^), (picog. mL−^1^), (ng. mL^−1^), (mg. mL^−1^), (pmol. L^−1^), (μg.L^−1^), (μg. mL^−1^), (μL/U/mL), (ug. dL^−1^), and (mg. mL^−1^).

### 3.5. Response of Ghrelin to Acute and Chronic Exercise Mode

#### 3.5.1. Response to Acute Exercise

Total ghrelin/ghrelin responses to acute exercise were assessed in 30 studies, while 1 study measured its response to both acute and chronic exercise [128].

##### Total Ghrelin/Ghrelin

Most of the included studies showed no change in circulating total ghrelin levels [19,23,24,108,109,110,111,113,114,115,116,123,124,126,127,128]. This lack of change was reported in ten studies involving a single short-duration exercise session [19,23,108,109,110,111,113,114,115,116] and six studies involving a long-term chronic exercise intervention (≥12 weeks) [24,123,124,126,127,128].

Total ghrelin levels remained stable, mostly after aerobic exercise [19,24,108,109,110,111,113,114,116,123,126,127,128], then resistance [115], combined [23], or intermittent [124] exercise. These studies included healthy normal weight [110,111,124,126], or overweight/obese [19,23,114,116], both normal weight and obese [115], trained [24,109,113,123,128] or untrained [127] individuals, and one study included both healthy subjects and GH-deficient patients [108].

Nine studies demonstrated reduced total ghrelin/ghrelin levels [11,22,117,118,119,120,121,129,130] after aerobic [22,117,118,119,120], resistance [11,129,130], or combined exercise [121] in normal weight [11,117,129], overweight/obese [22,121], or active individuals [118,119,120,130]. The decrease was observed after short [22,117,118,119,120,121], long [11,129], or very long-term training [130]. However, only four studies showed increased total ghrelin/ghrelin levels after short [122] or very long-term [20,21,131] aerobic training in trained subjects. Finally, two studies reported both unchanged and decreased or unchanged and increased total ghrelin/ghrelin levels after short [112], long, and very long-term [125] aerobic training in trained or healthy normal weight subjects depending on the participant’s sex (unchanged in females and decreased in males) or exercise intensity (100 W vs. 50 W), respectively [112,125].

##### Growth Hormone

Only 15 studies measured the GH responses to acute exercise [19,20,108,109,110,111,113,114,117,119,122,125,127,130,131], and most studies (n = 12) showed increased GH levels after exercise with varying durations and intensities [20,109,110,111,113,114,119,122,125,127,130,131]. However, three studies showed both increased and unchanged circulating GH depending on the participant’s health status [108] or the exercise intensity [19,117] after short-term acute training. An increase in GH levels was observed in seven short-term exercise studies [109,110,111,113,114,119,122], one long-term study [127], and four very long-term exercise studies [20,125,130,131].

#### 3.5.2. Response to Chronic Exercise

Total ghrelin/ghrelin responses to chronic exercise were assessed in 30 studies, while 1 study evaluated its response to both acute and chronic exercise [128].

##### Total Ghrelin/Ghrelin

Most studies demonstrated increased total ghrelin/ghrelin levels (n = 16) after aerobic [12,136,142,143,144,145,146,147,148,154], resistance training [29,150,152], combined [149,151], or intermittent training [28] among healthy normal weight [142,149,150], overweight/obese [28,136,143,144,145,146,147,151,152,154], or clinical overweight/obese with type 2 diabetes mellitus [148] or metabolic syndrome [29] and among children with precocious puberty [12]. The increase was mostly observed with long- and very long-term [12,142,143,144,145,146,147,148,149,150,151,152,154] rather than short-term [28,29,136] training programs.

Stable plasma ghrelin levels was shown in 11 studies using either short [25,132,133,134] or long-term [26,137,138,139,140,141] or very long-term [153] aerobic training [25,26,132,133,134,137,139,140], resistance [141], or combined training [138,153] in active [133], inactive [140], trained [132,134], healthy overweight/obese [25,137,139,141,153], and clinical obese with type 2 diabetes mellitus [148] or osteoarthritis [26].

However, four studies involving obese [10] and trained [27,128,135] individuals demonstrated a significant decrease in total ghrelin levels following both short- [27,135] and long-term [10,128] aerobic exercise. This reduction in ghrelin levels is thought to be linked to the body’s adaptation to energy expenditure during exercise [19,62,69,155,156,157,158], where changes in energy balance and fat stores may influence hunger signaling pathways. The mechanisms behind this reduction are complex and involve various factors, including alterations in adiposity, insulin sensitivity, and exercise-induced changes in the autonomic nervous system [159,160,161,162,163,164].

##### Body Mass, Body Mass Index, and Body Fat

Of the 31 included studies, only 2 did not measure changes in body composition after chronic training [10,132]. The majority of studies (n = 24) reported reduced body mass [143,146,154], body mass index [12,26,27,136,144,145,147,153], or body fat [25,28,29,134,135,137,139,140,141,142,150,151,152] after chronic training of varying intensities. The decrease was mostly observed with long- and very long-term [12,26,137,139,140,141,142,143,144,145,146,147,150,151,152,153,154] rather than short-term [25,27,28,29,134,135,136] training programs. Nevertheless, five studies found unchanged body composition training [128,133,138,148,149] after short training [133] and long [128,138,148,149] chronic training interventions. This suggests that prolonged exercise regimens may lead to more significant metabolic adaptations, including reductions in body fat and changes in hormonal signaling pathways that regulate hunger and satiety [165,166,167].

## 4. Discussion

The purpose of this systematic review is to examine changes in ghrelin levels in response to acute and chronic bouts of exercise. This review includes 61 studies that adopted exercise interventions with different modes, types, durations, and intensities. The results of this analysis found conflicting findings, with reports demonstrating increased, decreased, or unchanged total ghrelin/ghrelin levels in response to acute or chronic exercise.

### 4.1. The Effect of Acute Exercise on Total Ghrelin

Despite conflicting findings on the effects of acute exercise on circulating total ghrelin, over half of the studies assumed no significant impact [19,23,24,108,109,110,111,113,114,115,116,123,124,126,127,128], particularly following short-duration and aerobic protocols. This prevailing assumption suggests a potential bias or underestimation of exercise-induced hormonal dynamics, warranting more rigorous investigation. However, a recent systematic review [13] reinforces this notion, concluding that acute exercise does not significantly affect total ghrelin concentrations. This apparent stability could reflect a complex interplay of physiological mechanisms, such as the redistribution of blood flow away from ghrelin-producing tissues during exercise, as suggested by Hazell et al. [168]. Nevertheless, the heterogeneity in exercise modalities, intensities, and participant profiles limits the generalizability of these conclusions and highlights the need for further controlled investigations to clarify the underlying mechanisms.

Several studies have shown that acute exercise can suppress acylated ghrelin levels, its active isoform, particularly when the exercise is of moderate to high intensity. For example, Broom et al. [169] found that a 60-min run at 72% VO_2max_ significantly reduced plasma acylated ghrelin concentrations and hunger sensations compared to a resting period. Additionally, a meta-analysis by Schubert et al. [167] highlighted that acute exercise can influence appetite by suppressing acylated ghrelin levels, while simultaneously increasing levels of PYY, GLP-1, and PP, thereby contributing to changes in food and drink intake following exercise. These findings suggest that acute physical activity can modulate appetite through alterations in the levels of appetite-regulating hormones such as ghrelin, PYY, and GLP-1. However, it is important to note that most studies have measured only acylated ghrelin, and therefore the data for separately examining total, acylated, and des-acylated ghrelin are limited.

Although several reviewed studies showed an increase in total ghrelin levels following acute or prolonged aerobic exercise, the mechanisms underlying this response remain unclear. These findings suggest a more complex relationship between exercise and ghrelin regulation, which may vary based on several factors, such as exercise duration, intensity, and the metabolic state of the individual. The studies indicating increased ghrelin levels predominantly involved healthy normal-weight [112] or trained individuals [20,21,122,131], and these responses were observed after both short-term [112,122] and very long-term exercise protocols [20,21,131]. The increase in ghrelin in these contexts might reflect a peripheral marker of a negative energy balance, signaling the body’s metabolic response to energy needs during and after exercise [21]. This hypothesis is supported by the fact that ghrelin, often referred to as the “hunger hormone”, plays a crucial role in regulating appetite and energy homeostasis [6]. However, several questions remain unresolved. For example, why does ghrelin increase in some cases but decrease in others, and what role might individual differences (e.g., training status, body composition, and energy availability) play in this divergent response [143,154]. Current literature provides only partial explanations, and future research should aim to clarify the role of ghrelin as a regulator of energy balance in response to exercise. Studies should consider factors such as the time course of ghrelin release, the interaction between ghrelin and other appetite-related hormones like leptin and PYY [158,163], and the influence of various exercise modalities (e.g., aerobic vs. resistance training) [15,128]. Additionally, examining the differential responses based on participant characteristics, including metabolic status, age, and fitness level, could offer valuable insights into how exercise-induced changes in ghrelin contribute to appetite regulation and overall energy balance [170,171]. Ultimately, understanding the exact role of ghrelin in exercise-induced energy balance changes will provide key information for developing personalized exercise interventions aimed at weight management or improving metabolic health [64,172].

Several studies examining GH response to exercise have reported three distinct patterns: unchanged ghrelin with increased GH [19,108,109,110,111,113,114], concurrent increases in both ghrelin and GH [122], and decreased ghrelin levels alongside elevated GH [20,117,119,125,127,130,131]. These discrepancies suggest that ghrelin may not serve as a primary regulator of GH release during acute exercise, regardless of its duration. Instead, GH secretion in response to exercise appears to be primarily driven by hypothalamic neurohormones—GHRH and somatostatin—as supported by evidence in both human and animal models [36,37].

This review suggests that a single short-duration acute exercise session, particularly aerobic exercise, does not consistently alter ghrelin concentrations, even in the presence of elevated GH levels [19,108,109,110,111,113,114,122,125]. This supports the hypothesis that ghrelin plays a limited, if any, role in the regulation of GH during acute exercise [20,117,119,125,127,130,131]. However, this relationship remains incompletely understood, and the heterogeneity of findings highlights the need for further mechanistic studies [36,37]. It is also important to consider that ghrelin secretion is influenced by a variety of physiological factors, including nutritional status, the timing and composition of the preceding meal, circadian rhythms, and exercise intensity or duration [41,62,169,173]. In studies where these variables were not tightly controlled, any potential effect of acute exercise on ghrelin levels may have been obscured [143,161]. Future research should aim to isolate these confounding factors through rigorous experimental design to clarify the precise nature of the ghrelin/GH interaction under exercise conditions [170,172,174].

### 4.2. The Effect of Chronic Exercise on Total Ghrelin

Data on the total ghrelin response to training have shown controversial results, with most studies showing an increase and fewer reporting decreases or unchanged levels. Up to 51% of chronic training program studies demonstrated increased circulating total ghrelin [12,28,29,136,142,143,144,145,146,147,148,149,150,151,152,154]. The increase is mostly reported after aerobic [12,136,142,148,154], resistance [29,150,152], combined [149,151], or intermittent training [28]. Raised total ghrelin levels were especially seen in overweight/obese subjects [12,28,29,136,143,144,145,146,147,148,151,152,154].

Moreover, exercise training protocols that lead to increased total ghrelin are generally accompanied by lower body mass, body fat, or BMI [12,25,26,27,28,29,134,135,136,137,139,140,141,142,143,144,145,146,147,150,151,152,153,154]. Indeed, unchanged body composition occurred generally with unchanged plasma ghrelin level [128,133,138,148]. A recently published systematic review suggested that weight loss resulting from chronic training is the primary mechanism for increasing circulating ghrelin levels [13]. In light of this evidence, previous studies combining training programs with dietary intervention have reported an increase in total ghrelin only in participants who experienced significant weight loss [150]. Moreover, higher ghrelin levels are associated with greater weight loss [154]. Indeed, Mason et al. [154] showed that ghrelin increased by 8.4% in overweight/obese women who lost 5–10% of their body weight, compared to an increase of approximately 10% in those who lost ≥ 10% of their body weight, in response to diet plus exercise.

The exact process linking weight loss to increased circulating ghrelin levels is not yet fully elucidated, but several mechanisms have been proposed. One potential pathway involves metabolic adaptations: weight loss may enhance carbohydrate oxidation and reduce fat utilization independently of GH signaling [62,172]. In addition, increased GH and IGF-1 secretion following exercise mediate anabolic effects and indirectly promote ghrelin release [175,176,177]. Ghrelin also interacts with neuropeptide Y (NPY), a key hypothalamic regulator of long-term food intake, suggesting a neuroendocrine feedback loop in response to energy restriction [58]. Another potential mechanism linking weight loss to increased circulating ghrelin levels is the modulation of inflammation [12]. Evidence suggests that hypothalamic inflammation is a key contributor to ghrelin resistance [178]. Weight loss reduces systemic and central inflammatory markers, including pro-inflammatory cytokines such as IL-6 and TNF-α [12,16,179]. The reduction in inflammation may, in turn, help restore ghrelin sensitivity [174], thereby enhancing the hormone’s regulatory role in appetite and energy homeostasis. Furthermore, total ghrelin has been shown to increase with reduced oxidative stress [180,181,182]. Since regular exercise improves mitochondrial function and reduces reactive oxygen species production [180,183,184], this may represent an additional mechanism underlying ghrelin upregulation in response to chronic training.

Increased total ghrelin levels occurred particularly after long and very long-term training of low, moderate, or high intensity [12,142,143,144,145,146,147,148,149,150,151,152,154] than short-term moderate/intensive training [28,29,136]. Accordingly, total ghrelin release is more sensitive to training duration than training intensity, since long- and very long-term training [12,26,134,137,140,141,142,143,144,145,146,147,150,151,152,153,154] is more efficient in reducing body mass/fat, especially in obese people. The current review reinforces the approach that the effect of exercise training on ghrelin concentration depends on the volume of the training program [13,184]. In addition, the current review reports that increased total ghrelin is more sensitive to long and very long aerobic training, mainly in overweight/obese subjects.

In contrast, fewer studies have reported unchanged [25,26,132,133,134,137,138,139,140,141,153] or decreased [10,27,128,135] total ghrelin levels, after short- and long-term aerobic and resistance training, respectively. It has also been reported that plasma total ghrelin levels following exercise may be attenuated during periods of severe energy restriction [132], although the underlying mechanism remains unclear. Nutrition likely modulates the exercise-induced effect on postprandial ghrelin, depending on the body’s energy reserves. In particular, greater energy restriction appears to be associated with a more pronounced decrease in ghrelin levels [132,135].

The results of this analysis suggest that a single short-duration acute aerobic exercise session did not affect circulating total ghrelin levels, regardless of exercise intensity, participant characteristics, or GH secretion.

The study also revealed that long-term aerobic training may be an interesting modality to increase total ghrelin levels, mainly in overweight or obese individuals, through mechanisms associated with body composition improvements.

A key strength of this systematic review is that a comprehensive literature search strategy was used to identify all relevant research. This systematic review included studies with high heterogeneity in terms of participants and exercise characteristics. The study population included men and women, children, youth, adults, and elderly people of normal weight, overweight or obese, healthy or suffering from diseases. In addition, fitness levels varied from inactive, active, to athletes. The acute/chronic training intervention admitted low, moderate, or high-intensity aerobic, resistance, intermittent, or combined exercises of short, long, or very long duration. The training was combined with other interventions, incorporating diet, medication, or hormonal supplements. This large variability led to a challenging interpretation of the results and made it difficult to develop conclusive outcomes. It is unavoidable to highlight the limitations of this review. The included studies are limited to those that measured total ghrelin/ghrelin, as we excluded those that assessed both ghrelin isoforms, AG and DAG. Similarly, the sample size in most studies was small, suggesting that these studies may be underpowered to detect statistical significance. The restriction to the English language may be considered another limitation in this review.

A key methodological limitation identified across the studies is the inconsistency in reporting blood sample handling procedures, which could contribute to variability in ghrelin levels. Acylated ghrelin is highly unstable and requires specific stabilization techniques, such as protease inhibitors and rapid freezing, to ensure accurate measurement. Our analysis found that only a subset of studies provided detailed protocols for sample stabilization, while others did not provide sufficient detail or did not report methods at all. This lack of standardization likely contributes to the variability in reported plasma ghrelin levels following exercise interventions. As a result, we were unable to perform a quantitative analysis of the correlation between stabilization methods and study outcomes due to insufficient data.

Taken together, the evidence suggests that the effects of exercise on total ghrelin levels may differ significantly between healthy individuals and those with endocrine dysfunctions, especially when pharmacological treatments are involved. While several studies included in this review showed that chronic aerobic exercise can increase total ghrelin concentrations, particularly in overweight or obese participants, results from individuals with hormonal disorders were more heterogeneous. This variability may stem from the influence of prescribed medications (e.g., GH therapy) that interact with exercise-induced endocrine responses through different regulatory mechanisms. For instance, in GH-deficient patients, ghrelin dynamics may be more directly influenced by exogenous hormone replacement than by exercise itself. Therefore, the interpretation of results in these populations must be approached with caution, and future studies should aim to isolate the specific contribution of exercise in the presence of pharmacological confounders.

Our findings underscore the importance of exercise duration and intervention period in modulating total ghrelin levels. Short-duration acute exercise (<60 min) generally failed to produce consistent changes in ghrelin, regardless of intensity, likely due to insufficient energy deficit or limited hormonal adaptation time [169,173]. Conversely, long-term chronic exercise interventions (≥12 weeks), particularly those involving moderate-intensity aerobic exercise, were more likely to increase total ghrelin concentrations, especially in overweight or obese individuals [143,183]. This supports the hypothesis that chronic exercise may restore ghrelin sensitivity as part of broader metabolic adaptations, including improved insulin sensitivity, energy homeostasis, and body composition.

In this context, it is important to consider the interplay between ghrelin and other appetite-regulating gut peptides, particularly GLP-1. GLP-1, an incretin hormone, exerts anorexigenic effects by enhancing satiety and reducing gastric emptying, and has become a central focus in obesity treatment through GLP-1 receptor agonists [185,186]. While GLP-1 and ghrelin exert opposing effects on appetite, exercise appears to influence both pathways. Emerging evidence suggests that regular exercise may enhance GLP-1, contributing to appetite suppression and metabolic benefits [137]. Therefore, future research should explore how different training modalities simultaneously modulate ghrelin and GLP-1, and whether combining exercise with GLP-1 agonist therapy could provide synergistic effects in metabolic disease management.

## 5. Conclusions

The effect of short-duration, long-term, and very long-term acute and chronic exercise on ghrelin and GH levels remains inconsistent across studies. In addition to the direction of hormonal responses, the magnitude of change also varied considerably. Heterogeneous reporting—often using relative changes or non-standardized units—hinders direct comparisons and limits interpretability. Ghrelin responses ranged from modest increases of +20 to +200 pg/mL, with some interventions showing elevations exceeding 300 pg/mL. Lower-intensity, prolonged aerobic exercise (e.g., 50 W cycling or ~50–60% VO_2max_) typically induced moderate increases in ghrelin, whereas higher-intensity or resistance protocols (≥70% VO_2max_, sprints) often resulted in stable or reduced levels. These differences may reflect variations in sympathetic activity, gastrointestinal blood flow redistribution, or the suppression of appetite-regulating hormones.

In contrast, GH showed more consistent intensity-dependent increases, with post-exercise levels rising from approximately 2 to over 10 ng/mL, particularly during high-intensity protocols exceeding 75% VO_2max_ or approaching the lactate threshold. Moderate-intensity sessions (50–60% VO_2max_) produced smaller or negligible changes. These findings suggest a positive relationship between exercise intensity and GH secretion, likely mediated by hypothalamic GHRH stimulation, although influenced by individual fitness, energy status, and sampling timing.

To better characterize these dose–response relationships, future studies should adopt standardized protocols for exercise intensity, duration, and hormone measurement.

## Figures and Tables

**Table 1:** Effects of short-term (<60 min) exercise on total ghrelin.

Reference	Study Population	Intervention	Results (Ghrelin and Growth Hormone (GH))
Short-term (<60 min) exercise with stable plasma ghrelin levels			
Dall et al. [108]	Eight healthy males (age, 40.8 ± 2.9 years; BMI, 23.6 ± 0.5 kg/m^2^). Eight hypopituitary males with GH deficiency (age, 40.8 ± 4.7 years; BMI, 29.2 ± 0.8 kg/m^2^).	Submaximal aerobic exercise For 45 min At 65–70% of HR_max_.	Ghrelin levels did not change significantly (*p* > 0.05). GH concentrations increased after 45 min of exercise (11.43 ± 3.61 µg/L). Infusion of GH in the patients resulted in a peak level after 45 min. GH did not increase after exercise without GH infusion (9.77 ± 2.40 with GH vs. 0.11 ± 0.07 µg/L without GH).
Kraemer et al. [109]	Six well-trained males (age, 27.7 ± 3.20 years; BM, 72.0 ± 4.6 kg).	Progressively intense exercise on a treadmill, including four exercise intensities: 60%, 75%, 90%, and 100% of VO_2max_. Blood samples were collected before and after 15 and 30 min.	Ghrelin levels did not change significantly. Intense running (75% to 100% of VO_2max_) increased IGF-1 and GH levels.
Schmidt et al. [110]	Eight healthy young males (age, 29.9 ± 1.9 years; BMI, 22.2 ± 0.5 kg/m^2^).	Treadmill exercise at 50%, 70%, and 90% of VO_2max_ for 10–20 min on different days.	Ghrelin plasma concentrations remained unchanged (−9% at 50% VO_2max_, −10.65% at 70% VO_2max_, −2.64% at 70% VO_2max_, *p* > 0.05). GH increased significantly after 40 min at 50% VO_2max_ and after 20 min at 70 and 90% VO_2max_. GH peak concentrations were 5.8 ± 2.3 ng/mL, 12.0 ± 3.2 ng/mL, and 9.8 ± 4.7 ng/mL, respectively.
Zoladz al. [111]	Eight healthy non-smoking men (age, 23.0 ± 0.5 years; BM, 71.9 ± 1.5 kg; BMI, 22.42 ± 0.49 kg/m^2^).	Two incremental cycling tests: one in the fed state until exhaustion, and one after overnight fasting until reaching 150 W, with 30 W increases every 3 min.	Pre-exercise leptin and ghrelin levels did not differ between fed and fasted states. Plasma GH was significantly higher at 90 W in the fed state (*p* = 0.016). At 150 W, GH reached 14.85 ± 4.67 ng/mL (fed) and 12.83 ± 3.62 ng/mL (fasted).
Erdmann et al. [112]	Seven healthy males and females (age, 24.4 ± 0.6 years; BMI, 21.4 ± 0.8 kg/m^2^).	Bicycle exercise for 30 min at 100 W, below the aerobic/anaerobic threshold. In a second group, 7 subjects cycled at 50 W for 30, 60, and 120 min, respectively.	Ghrelin levels increased during exercise at 50 W, rising by 50–70 pg/mL above baseline after 30, 60, and 120 min. However, during higher intensity cycling at 100 W, ghrelin levels remained unchanged, and hunger/satiety ratings, food intake, and postprandial ghrelin suppression were like the control group, showing no significant differences.
		Diet (40–50% carbohydrate, 15–25% protein, and 30–40% fat).	
Jürimäe et al. [113]	Nine elite male rowers (age, 20.1 ± 3.7 years; BMI, 20.1 ± 3.7 kg/m^2^; BM, 89.6 ± 4.6 kg; %body fat, 9.9 ± 2.5%).	Single-scull rowing was performed for 15 min below and above the individual anaerobic threshold.	Above anaerobic threshold
			Plasma ghrelin concentration did not increase significantly (from 802 ± 181 to 808 ± 196 pg/mL, +0.74%, *p* > 0.05). GH significantly increased (from 2.6 ± 4.4 to 72.6 ± 19.2 ng/mL, +2692.3%, *p* < 0.05).
			Below anaerobic threshold
			Plasma ghrelin concentration did not increase significantly (from 791 ± 214 to 794 ± 170 pg/mL, +0.37%, *p* > 0.05). GH significantly increased (from 4.1 ± 4.8 to 41.9 ± 28.4 ng/mL, +922%, *p* < 0.05).
Marzullo et al. [114]	Eight obese males (BMI, 33.7 ± 1.5 kg/m^2^).	Cycling exercise, at 20 W, increased by 20 W every 4 min until exhaustion.	Plasma ghrelin concentration did not increase significantly (from 3053 ± 315 to 2929 ± 345 pg/mL, −4.06%, *p* > 0.05). GH increased significantly (from 1.3 ± 0.96 to 5.5 ± 1.6 µg/L, +323.1%, *p* < 0.01)
Thomas et al. [115]	Five class 1 obese men (age, 21.6 ± 2.5 years; BMI range, 30.00–34.99 kg/m^2^; BM, 97.8 ± 8.58 kg; %body fat, 34.7 ± 2.95%). Five class 2 obese men (age, 20.0 ± 1.4 years; BMI range, 35–39.99 kg/m^2^ BM, 120.8 ± 10.49 kg; %body fat, 40.5 ± 5.82%). Nine lean men (age, 20.1 ± 2.1 years; BMI range, 35–39.99 kg/m^2^; %body fat, 14.7 ± 3.54%).	Resistance exercise protocol: 6 exercises, 3 sets of 10 repetitions at 85–95%, 10 repetition maximum with 120- and 90-s rest periods.	The obese 2/3 class group had significantly greater ghrelin levels than the lean group (*p* = 0.009) and the obese class 1 group (*p* = 0.002). Higher GH was associated with lower ghrelin in lean individuals.
Crabtree and Blannin, [116]	Sixteen overweight participants: ten men and six women (age, 50.1 ± 11.6 years; BMI, 28.9 ± 4.2 kg/m^2^).	Treadmill walking (cold trial and neutral trial) for 45 min at 60% of VO_2max_ in a randomized, counterbalanced design.	Total ghrelin concentration was significantly greater during walking in the cold versus those during walking in the neutral condition (*p* < 0.05).
Ouerghi et al. [19]	Seven inactive overweight men (age, 36.4 ± 4.35 years; BMI, 28.3 ± 1.66 kg/m^2^).	Two sessions of cycling exercise at 7-day intervals, each session lasting 20 min at 60% or 80% of peak aerobic power.	Ghrelin was unchanged in both groups. GH increased significantly at the end of exercise (from 0.12 ± 0.09 to 1.08 ± 0.73 ng/mL, +800%, *p* = 0.004) and 30 min later (from 0.12 ± 0.09 to 0.60 ± 0.36 ng/mL, +400%, *p* = 0.035) the session at 80% of peak aerobic power.
Tobin et al. [23]	Twenty-four overweight/obese subjects, twelve men and twelve women, matched for age: 32.3 ± 2 vs. 36.8 ± 2 years, and BMI: 28.1 ± 1.2 vs. 29.0 ± 1.5 kg/m^2^.	(1) Aerobic exercise at 65–70% of age-predicted HR_max_ for 45 min. (2) Twelve resistance exercises, each performed as one set to failure. (3) Sedentary control.	Total ghrelin showed no significant differences between men and women after acute exercise (all *p* > 0.05).
Short-term (<60 min) exercise with lower plasma ghrelin concentrations			
Toshinai et al. [117]	Five inactive normal weight healthy men (age, 26 ± 0.5 years; BMI, 23.3 ± 0.2 kg/m^2^).	Incremental endurance exercise for 10 min, including 4 conditions: Half of the lactate threshold. Lactate threshold. Onset of blood lactate accumulation. Above the onset of blood lactate accumulation.	Ghrelin levels decreased significantly after incremental exercise. GH did not change after exercise at half the lactate threshold intensity incremental exercise (*p* > 0.05). GH increased after exercise at an intensity at the lactate threshold intensity (*p* < 0.05). GH increased after exercise at an intensity at the onset of blood lactate accumulation (*p* < 0.001). GH increased exercise at an intensity above the onset of blood lactate accumulation (*p* < 0.001).
Stokes et al. [119]	Seven healthy, active men (age, 26.0 ± 3.0 years; BMI, 24.9 ± 3.5 kg/m^2^).	In two exercise trials, participants performed a single 30-s sprint on a cycle ergometer against a resistance equivalent to 7% (FAST) or 9% (SLOW) of their body mass. A control group rested in the laboratory.	Total ghrelin concentrations decreased significantly after the sprint, with values lower at 30 min of recovery compared to pre-exercise (FAST: from 620 ± 190 to 490 ± 160 pg/mL, −21.0%, *p* < 0.001; SLOW: from 590 ± 150 to 470 ± 130 pg/mL, −20.3%, *p* < 0.001). GH concentrations increased in both exercise trials and were greater in the FAST than in the SLOW trial.
Kelly et al. [120]	Ten physically active men (age, 21.4 ± 1.3 years; BMI, 23.94 ± 2.1 kg/m^2^).	Treadmill running for 45 min at intensity of 70% of VO_2peak_. In a randomized, counterbalanced design, males completed three trials: exercise when hydrated (0–1% body mass), exercise when dehydrated (−1% to −2% body mass), and a hydrated resting control.	Exercise performed in a dehydrated state resulted in significantly lower concentrations of ghrelin compared with control (*p* = 0.045) and hydrated exercise conditions (*p* = 0.014).
Halliday et al. [121]	Twenty-four physically inactive and overweight/ obese adults: twelve men and twelve women (age, 35 ± 2 years; BMI, 28.5 ± 0.9 kg/m^2^; %body fat, 35 ± 2%).	Three conditions were initiated 35 min after breakfast: Aerobic exercise (walking at 65–70% HR_max_ for 45 min). Acute bout of resistance exercise (12–15 repetitions for 12 exercises). Sedentary control.	Ghrelin was lower after resistance exercise (131 ± 4 pg/mL) than after aerobic exercise (144 ± 7 pg/mL) (*p* = 0.006).
Li et al. [22]	Fourteen obese: seven men and seven females (age, 20.6 ± 1.5 years; BMI, 31.1 ± 2.0 kg/m^2^, BM; 88.7 ± 15.1 Kg; %body fat 38.8 ± 3.7%).	The participants were randomized into three groups performing: (1) Moderate-intensity continuous exercise without blood flow restriction, 60% VO_2max_, 200 kJ. (2) Moderate-intensity continuous exercise with blood flow restriction, 60% VO_2max_, 200 kJ. (3) Control session without exercise.	No significant differences in total ghrelin were detected before exercise. Immediately after exercise, total ghrelin in the (1) and (2) groups differed significantly from the (3) group (*p* < 0.05). One hour after exercise, total ghrelin in the (2) and (1) groups remained significantly different from the (3) group (*p* < 0.05). Total ghrelin in the (2) group was significantly lower than in the (1) group (*p* < 0.05).
Short-term (<60 min) exercise with higher plasma ghrelin levels			
Erdmann et al. [112]	Seven normal-weight men and women (age, 24.4 ± 0.6 years; BMI, 21.4 ± 0.8 kg/m^2^).	Bicycle exercise on an ergometer for 30 min at 50 W, below the aerobic/anaerobic threshold.	In the control group, plasma ghrelin postprandial declined at 150 min (from 488.6 ± 80.8 pg/mL to 325.4 ± 54.1 pg/mL, −33.4%, *p* < 0.05). During 30 min of exercise at 50 W, ghrelin increased (from 520.4 ± 82.7 pg/mL to 566.6 ± 86.2 pg/mL, +8.9%, *p* < 0.05), then decreased to 386.8 ± 58.5 pg/mL, −25.7%, *p* < 0.05 at 135 min. At 100 W, ghrelin remained unchanged (485.7 ± 94.7 pg/mL to 473.4 ± 101.8 pg/mL, −2.47%, *p* > 0.05).
		Diet (40–50% carbohydrate, 15–25% protein, and 30–40% fat).	
Jürimäe et al. [122]	Eight male rowers (age, 21.3 ± 2.8 years; BM, 97.4 ± 67.4 kg).	Maximal rowing ergometer test for 20 min at 81% of VO_2max_.	Ghrelin significantly increased immediately after the exercise (+24.4%, *p* < 0.05) and was not significantly different than baseline after 30 min of recovery. GH increased from 0.9 ± 0.6 to 72.1 ± 9.5 μL/U/mL, +7902.2%, *p* < 0.05.
Long-term (≥60 min), exercise with stable plasma ghrelin levels			
Burns et al. [123]	Eighteen healthy trained: nine men and nine women (age, ♂24.5 ± 1.3 years, ♀25.1 ± 1.2 years; BM, ♂74.03 ± 4.20 kg, ♀63.57 ± 2.55 kg; BMI, ♂23.4 ± 1.0 kg/m^2^ ♀22.5 ± 0.8 kg/m^2^; %body fat, ♀16.9 ± 1.7%, ♀28.3 ± 1.2%).	Treadmill run for 60 min at 73.5% of VO_2max_.	Ghrelin concentrations did not differ between trials (from 1387.6 ± 254.5 to 1380.6 ± 224.9 pg/mL, −0.5%, *p* > 0.05).
Martins et al. [124]	Twelve healthy, normal-weight volunteers (six males and six females) (age, 25.9 ± 4.6 years; BMI, 22.0 ± 3.2 kg m^2^).	Ergometer intermittent cycling for 60 min at 65% of HR_max_.	No significant effect of exercise was observed on postprandial levels of ghrelin (*p* > 0.05).
Sartorio et al. [125]	Group 1: nineteen healthy males and eighteen females (age, 25 ± 6.7 years). Group 2: four healthy males (age, 28.2 ± 7.2 years).	Group 1: Aerobic exercise (treadmill running) for 60–90 min at 80% of VO_2max_. Group 2: Two consecutive 30-min cycling sessions at 80% VO_2max_ with different time intervals (2 and 6 h) between bouts on two separate days.	Group 1: In males, ghrelin levels significantly decreased (from 1506.4 ± 859 to 1254.8 ± 661.7 pg/mL, −16.7%, *p* < 0.05), while no significant changes were observed in females. GH levels increased post-training (*p* < 0.0001), with no sex differences. Group 2: No significant changes in ghrelin levels were observed during or after the two bouts at different intervals. GH levels increased after the first exercise bout (peak: 26.8 ± 11.2 and 17.3 ± 3.5 ng/mL, *p* < 0.005). Peak GH after the second bout (4.3 ± 1.6 ng/mL) was lower (*p* < 0.01) after a 2-h interval, while GH responsiveness recovered after the 6-h interval (11.9 ± 3.3 ng/mL). GH responses to prolonged exercise (60–90 min) were linked to changes in ghrelin levels only in males, while repeated shorter bouts (30 min) with marked GH responses did not affect ghrelin concentrations.
Hagobian et al. [126]	Eighteen healthy overweight/obese individuals, nine men and nine women (age, ♂26.8 ± 11.8 years, ♀23.3 ± 8 years; BMI, ♂25.7 ± 2.3 kg/m^2^; ♀28.0 ± 3.5 kg/m^2^).	Treadmill running at 50–65% of VO_2peak_. Four bouts with energy added to the baseline diet to maintain energy balance and four bouts without added energy to induce energy deficit.	In men, no significant changes in ghrelin levels were observed. In women, ghrelin was higher after both the energy deficit (+32%) and energy balance (+25%) conditions, with the change from baseline being significantly higher in women compared to men (*p* < 0.05).
Shiiya et al. [127]	Nine healthy males (age, 25.2 ± 0.5 years; BMI, 22.6 ± 0.4 kg/m^2^).	Cycling exercise for 60 min at 50% of VO_2max_.	Plasma ghrelin levels decreased significantly during cycling (45–60 min) and tended to decline post-exercise (15–30 min, *p* ≈ 0.06). Deacyl and total ghrelin remained unchanged. GH significantly increased during exercise (*p* < 0.01) and decreased at 90 min.
Plinta et al. [128]	Fifty healthy young female professional basketball and handball players (age, 21 ± 2.4 years; BMI, 22.1 ± 1.8 kg/m^2^).	Three-month period of moderate aerobic training (pulse 140–160/min) or intensive aerobic training (pulse > 170/min).	Plasma ghrelin levels significantly decreased after long-term moderate aerobic exercise (921 ± 300 vs. 575 ± 572 pg/mL, −37.6%, *p* < 0.001), while they remained unchanged following short-term moderate aerobic exercise or intensive fitness and speed exercise.
Laursen et al. [24]	Eleven recreationally trained males (age, 25 ± 4 years; BM, 79.4 ± 13.5 kg).	Three 1-h cycling bouts at 60% W_max_ in hot (33 °C), cold (7 °C), and room temperature (20 °C), followed by a 3-h recovery at room temperature.	Total and acylated ghrelin levels remained unchanged post- and 3 h post-exercise, regardless of ambient temperature (*p* > 0.05).
Long-term (≥60 min), exercise with lower plasma ghrelin levels			
Ballard et al. [129]	Twenty-one healthy males (age, 20 ± 1.8 years; BMI, 24.8 ± 3.3 kg/m^2^).	Resistance exercise for 80 min at 55–70% of 1 RM and without carbohydrate supplementation.	Plasma ghrelin declined during and 110 min after exercise (*p* < 0.05). Plasma ghrelin increased in rest.
Liu et al. [11]	Eleven healthy young men (age, 23 ± 2 years; BMI, 22 ± 2 kg/m^2^).	Sixty minutes of moderate-load resistance exercise (4 sets of 8 repetitions at 85% 8 RM). Low-load resistance exercise (4 sets of 15 repetitions at 45% 8 RM).	Ghrelin concentrations were significantly lower immediately after both moderate and low-load exercise compared to control (*p* < 0.05).
Very long-term (≥90 min), exercise with lower plasma ghrelin levels			
Ghanbari-Niaki, [130]	Fourteen volunteer male physical education students (age, 20.5 ± 0.5 years; BM, 78.25 ± 5.01 kg; BMI, 25.27 ± 1.18 kg/m^2^).	Circuit resistance training for 180 min (10 exercises, three circuits, with 8–12 repetitions, at 60% of 1 RM).	Plasma ghrelin showed a significant decrease immediately after the exercise (*p* < 0.05) and increased significantly 24 h following the exercise (*p* < 0.05). GH showed a significant increase immediately after exercise (*p* < 0.01) and returned to pre-exercise values.
Sartorio et al. [125]	Group 1: Nineteen elite male athletes and eighteen elite female athletes (age, 25 ± 6.7 years; BMI, 25 ± 6.7 kg/m^2^). Group 2: Four elite male athletes (age, 28.2 ± 0.2 years; BMI, 25 ± 6.7 kg/m^2^).	Group 1: One 60–90 min training session at approximately 80% of VO_2max_; Group 2: Two consecutive 30-min cycling sessions at 80% of VO_2max_ at different intervals between workouts (2 and 6 h) on two different days.	Group 1: In males, ghrelin significantly decreased after the training session (from 1506.4 ± 859 to 1254.8 ± 661.7 pg/mL, −16.7%, *p* < 0.05), while no significant changes were found in females. GH levels increased after the training session (*p* < 0.0001), with no differences between males and females. Group 2: Ghrelin levels remained unchanged during or after the two exercise bouts at different time intervals. GH levels significantly increased after the first exercise bout (peak: 26.8 ± 11.2 and 17.3 ± 3.5 ng/mL). After the second bout, peak GH was lower (4.3 ± 1.6 ng/mL) at a 2-h interval (*p* < 0.01) but recovered after the 6-h interval (11.9 ± 3.3 ng/mL).
Very long-term (≥90 min), exercise with higher plasma ghrelin levels			
Christ et al. [131]	Eleven healthy, endurance-trained male athletes (age, 31.4 ± 1.7 years; BMI, 22.6 ± 0.5 kg/m^2^).	Aerobic exercise test on a cycloergometer for 180 min at 50% of W_max_. High-fat (HF) or low-fat (LF) diet.	Ghrelin significantly increased after the LF diet compared to the HF diet (*p* < 0.03). No differences in ghrelin levels were observed during exercise, but post-exercise ghrelin was significantly higher after the LF diet. GH levels were not significantly different between the LF and HF diets during the exercise session.
Jürimäe et al. [21]	Nine national-level male rowers (age, 20.1 ± 1.5 years; BM, 81.0 ± 5.0 kg; %body fat, 10.8 ± 3.3%).	Rowing training session for 120 min (distance = 20.7 ± 1.4 km; HR = 133 ± 4 bpm at intensity of 80.2 ± 1.6% of the HR turn point) followed by a 30-min rest.	Ghrelin concentration increased (from 780.6 ± 207.4 to 876.2 ± 207.3 pg/mL, +12.2%, *p* < 0.05) 30 min after exercise.
Russel et al. [20]	Twenty-one endurance-trained runners: eleven men (age, 27 ± 9 years; BM, 69.8 ± 4.9 kg, BMI 21.9 ± 1.5 kg/m^2^, %body fat, 12.8 ± 2.4%) and ten women (age 29 ± 7 years; BM, 56.2 ± 4.9 kg, BMI, 21.0 ± 1.1 kg/m^2^, %body fat 20.6 ± 2.3%).	Intense endurance running by a 10-km time trial on a treadmill for 90 min at 62 ± 5% of VO_2max_.	Ghrelin was significantly higher after exercise (from 823 ± 94 to 976 ± 94 pg/mL, +18.6%, *p* < 0.0001). GH significantly increased after exercise (from 2.7 ± 6.0 to 23.8 ± 16.8 μg·mL^−1^, +781%, *p* < 0.001).

Short-term exercise is defined as a single exercise session lasting less than 60 min. All interventions in this table refer to acute exercise (i.e., a single bout). Data are expressed as mean ± SD. BM, body mass; BMI, body mass index; GH, growth hormone; HR, heart rate; HR_max_, maximum heart rate; IGF-1, Insulin-like growth factor-1; RM, repetition maximum; VO_2max_, maximal oxygen uptake; VO_2peak_, peak of oxygen consumption; W_max_, Wattage maximum.

**Table 2:** Effects of chronic exercise training programs on total ghrelin according to program duration (short-term <12 weeks; long-term ≥12 weeks).

Reference	Study Population	Intervention	Results (Ghrelin and Body Mass/Body Fat /BMI)
Short-term chronic exercise programs (<12 weeks) with stable plasma ghrelin levels			
Rämson et al. [132]	Eight trained male rowers (age, 20.2 ± 1.6 years; BM, 81.0 ± 5.4 kg).	Week 1: Maintain previous training volume (10 h/week). Week 2: Increase training load by 50%. Week 3: Increase training load by 10–15% (individualized). Week 4: Decrease training volume back to week 1 level. Training Breakdown: 80% low-intensity rowing; 10% low intensity running/cycling; 10% strength endurance training (40–50% 1 RM, 30–50 reps). Frequency: 6 training days/week, 1 recovery day.	Week 1: Ghrelin increased from 780.5 ± 221.7 to 842.1 ± 216.5 pg/mL, +7.9% (*p* > 0.05). Week 2–3: Ghrelin increased from 819.4 ± 194.6 to 822.9 ± 215.5 pg/mL, +0.43% (*p* < 0.05). Week 4: Ghrelin increased from 755.6 ± 140.9 to 851.3 ± 229.1 pg/mL, +12.7% (*p* > 0.05).
Hedayati et al. [133]	Twenty-seven female students (age, 22.2 ± 1.54 years; BM, 52.6 ± 3.2 Kg, BMI, 20.76 ± 1.86 kg/m^2^, %body fat, 21.1 ± 1.6%).	Subjects performed circuit resistance training with 40% and 80% of 1 RM for 4 weeks.	At 40% of 1 RM: Ghrelin increased from 414 ± 154 to 446 ± 186 pg/mL, +7.73% (*p* > 0.05). The %body fat decreased from 21.2 ± 2.4% to 20.4 ± 2% (*p* > 0.05). At 80% of 1 RM: Ghrelin increased from 397 ± 195 to 451 ± 142 pg/mL, +13.60% (*p* > 0.05). The %body fat did not change from 20.6 ± 2.3% to 20.6 ± 2.3% (*p* > 0.05).
Rosenkilde et al. [134]	Six older men cyclists (age, 61 ± 3 years; BM, 52.6 ± 3.2 Kg, BMI, 24.46 ± 0.9 kg/m^2^; %body fat, 21.1 ± 1.6%).	Cycling (2706 km) for 14 days.	Total ghrelin decreased from 867 ± 516 to 824 ± 497 pg/mL, −4.95% (*p* > 0.05). Body fat decrease from 14.0 ± 1.5 kg to 11.8 ± 1.1 kg (*p* = 0.02).
Ahmadi et al. [25]	Thirty elderly men (age range 60–70 years; BMI range, 25–30 kg/m^2^).	Eight weeks of aerobic training at 50–60% of HR_max_, for 50–60 min/day.	Ghrelin increased from 640 ± 110 to 710 ± 20 pg/mL, +10.9% (*p* > 0.05). The %body fat decreased from 24.31 ± 3.39% to 21.43 ± 2.94% (*p* = 0.001).
Short-term chronic exercise programs (<12 weeks) with lower plasma ghrelin levels			
Rämson et al. [135]	Twelve trained male rowers (age 22.2 ± 3.4 years; BMI, 23.95 ± 2.4 kg/m^2^).	At 50% resistance and 50% endurance rowing, cycling, or running training. Week 1: 10 h/week. Week 2–3: 15 h–20 h/week. Week 4: 10 h/week.	Week 1: Ghrelin increased from 973.46 ± 183.4 to 980 ± 300.2 pg/mL, +0.71% (*p* > 0.05). Week 2–3: Ghrelin decreased from 980 ± 300.2 to 873.35 ± 198.61 pg/mL, −10.88% (*p* < 0.05). Body fat decreased from 11.4 ± 6.2 to 9.3 ± 5.5 kg (*p* < 0.05).
Cho et al. [27]	Forty women cadets (age 22 ± 28 years; BMI 24.1 ± 2.7 kg/m^2^).	Training course for 8 weeks.	Ghrelin decreased from 1100 ± 100 to 1000 ± 100 pg/mL, −9.1% (*p* < 0.01). BMI decreased from 24.1 ± 2.7 to 22.4 ± 2.2 kg/m^2^ (*p* < 0.01).
Short-term chronic exercise programs (<12 weeks) with hyperplasma ghrelin levels			
Azizi et al. [136]	Twenty-four inactive students (age 27.56 ± 0.48 years; BMI 32.68 ± 0.84 kg/m^2^).	Eight weeks of aerobic training for 60 min at 65–85% of HR_max_.	Ghrelin increased from 321 ± 65 to 417 ± 72 pg/mL, +29.90% (*p* = 0.0001). BMI decreased from 32.94 to 31.65 kg/m^2^ (*p* < 0.05).
Tremblay et al. [29]	Seventy-one overweight adults/elderly females and males with metabolic syndrome (age range 50–70 years). Group 1: BMI 32.68 ± 0.84 kg/m^2^. Group 2: BMI, 34.4 ± 4.2 kg/m^2^. Group 3 BMI, 33.9 ± 4.0 kg/m^2^.	Resistance training for 90 min/week for 3 weeks. Group 1: High resistance/moderate endurance, 65–85% of 10 RM for resistance training and 30% VO_2peak_ for endurance training. Group 2: Moderate resistance/high endurance intensity, 30% of 10 RM for resistance training and 70% VO_2peak_ for endurance training. Group 3: moderate resistance/endurance, 30% of 10 RM for resistance training/30% VO_2peak_ for endurance training.	Ghrelin was significantly increased after day 21 and month 3 (*p* < 0.001). Group 1: Body fat decreased from 27.7 ± 7.6 kg to 24.9 ± 7.1 kg (*p* < 0.05). Group 2: Body fat decreased from 32.2 ± 7.7 to 29.3 ± 7.3 kg (*p* < 0.05). Group 3: Body fat decreased from 32.3 ± 7.5 to 30.1 ± 7.3 kg (*p* < 0.05).
Liao et al. [28]	Sixteen obese children (age 12.74 ± 1.94 years; BMI, 27.74 ± 3.33 kg/m^2^).	Six weeks of moderate, high-intensity interval and resistance training at 50–60% of HR_max_ or 80–90% of HR_max_; 12–15 RM. Diet.	Ghrelin was significantly enhanced after 6 weeks (*p* < 0.05). The %body fat decreased from 40.00 ± 4.99% to 32.95 ± 4.66% (*p* < 0.001).
Long-term training (≥12 weeks) with stable plasma ghrelin levels			
Martins et al. [137]	Twenty-two overweight/ obese sedentary females and males (age range 56–70 years; BMI, 31.3 ± 2.3 kg/m^2^).	Twelve weeks of moderate, high-intensity interval treadmill walking or running at 74% of HR_max_.	Ghrelin increased from 2074 ± 912.4 to 2371 ± 1022 pg/mL, +14.3% (*p* > 0.05). The %body fat decreased from 35.3 ± 5.6% to 33.5 ± 5.9% (*p* < 0.001).
Kadoglou et al. [138]	Ninety females and males with type 2 diabetes mellitus (age 36.9 ± 8.3 years; BMI, 31.3 ± 2.3 kg/m^2^). Group 1: BMI, 32.55 ± 3.11 kg/m^2^, resistance training. Group 2: BMI, 31.55 ± 3.11 kg/m^2^, aerobic training. Group 3: BMI, 31.91 ± 2.93 kg/m^2^, aerobic exercise + resistance training. Group 4: BMI, 32.1 ± 2.95 kg/m^2^, control.	Twenty-four weeks of aerobic, resistance, and combined training for 60 min at 60–75% of HR_max_ or 60–80% of 1 RM.	Subgroup analysis showed no significant effects on serum ghrelin levels (*p* = 0.228). Group 1: %Body fat decreased—0.12 ± 0.06%. Group 2: %Body fat decreased—0.66 ± 0.21%. Group 3: %Body fat decreased—1.97 ± 0.55% (*p* < 0.005).
Rosenkilde et al. [139]	Fifty-three sedentary, overweight males (age range 27–30 years). Group 1: BMI, 27.6 ± 1.4 kg/m^2^. Group 2: BMI, 28.6 ± 1.8 kg/m^2^. Group 3: Control BMI, 28.0 ± 2.3 kg/m^2^.	Twelve weeks of aerobic training at 20–60 %VO_2max_ for 2984.6 ± 132.6 min. Group 1: High training dose, 60 min. Group 2: Moderate training dose, 30 min.	Group 1: Ghrelin increased from 648 ± 80 to 661 ± 84 pg/mL, +2% (*p* > 0.05). Body fat decreased from 27.4 ± 4.2 to 23.7 ± 3.7 kg (*p* < 0.001). Group 2: Ghrelin increased from 698 ± 209 to 714 ± 208 pg/mL, +2.29% (*p* > 0.05). Body fat decreased from 30.0 ± 4.6 to 25.8 ± 5.1 kg (*p* < 0.001). Group 3: Ghrelin increased from 674 ± 136 to 704 ± 122, +4.45% (*p* > 0.05). Body fat did not change from 29.0 ± 6.0 to 25.8 ± 5.1 kg (*p* > 0.05).
Gibbons et al. [140]	Thirty-two inactive males (age range 18–55 years). Group 1: BM, 83.5 ± 3.1 kg. Group 2: BM, 90.4 ± 2.7 kg. Group 3: BM, 93.1 ± 3.6 kg.	Twelve weeks of resistance training at 70% of HR_max_.	Ghrelin does not change (*p* > 0.05). Group 1: %Body fat decreased from 33.1 ± 2.5 to 29.0 ± 3.1 kg (*p* < 0.01). Group 2: %Body fat did not change 35.5 ± 3.3 to 35.0 ± 3.5 kg (*p* > 0.05). Group 3: %Body fat increased from 35.1 ± 2.2 to 40 ± 7.2% (*p* < 0. 01).
Elerian et al. [141]	Twelve obese females and males (age 34.75 ± 4.18 years; BMI, 37.3 ± 2.6 kg/m^2^).	Fourteen weeks of 50 min/day, 80% of 1 RM. Diet (1200–1800 kcal/j).	Ghrelin did not change from 4.4 ± 0.53 to 4.2 ± 0.35 mg/mL, −4.6% (*p* > 0.05). %Body fat decreased from 45.4 ± 5.2% to 40 ± 7.2 % (*p* < 0.005).
Fico et al. [26]	Thirty-nine obese females and males with osteoarthritis (age 59 ± 1 years). Group Cycling: BMI, 32.5 ± 2.0 kg/m^2^. Group Swimming: BMI, 33.1 ± 1.6 kg/m^2^.	Twelve weeks of cycling or swimming training, 20–45 min at 40–70% of HRr.	Ghrelin did not change (*p* > 0.05). Group Cycling: BMI decreased from 33.1 ± 1.6 to 32.2 ± 1.5 kg/m^2^ (*p* < 0.05). Group Swimming: BMI decreased from 32.5 ± 2.0 to 32.0 ± 2.0 kg/m^2^ (*p* < 0.05).
Long-term training (≥12 weeks) with lower plasma ghrelin levels			
Plinta et al. [128]	Fifty female professional basketball and handball players (age 21 ± 2.4 years); BMI, 22.1 ± 1.8 kg/m^2^).	Twelve weeks of moderate aerobic training or intensive aerobic training for 120 min at 140–170 bpm.	After 12 weeks of moderate aerobic training, ghrelin decreased from 921 ± 300 to 575 ± 572 pg/mL, −37.6% (*p* < 0.001). BM did not change (from 65.9 ± 7.1 kg to 65.5 ± 7.0 kg, *p* > 0.05). After 12 weeks of intensive aerobic training, ghrelin increased from 6.09 ± 5.62 to 6.89 ± 5.62 ng/mL, +13.1% (*p* > 0.05). BM did not change (*p* > 0.05).
Alyar et al. [10]	Sixty-two obese (BMI ≥ 30 kg/m^2^) and forty-eight healthy controls (BMI, range 18.50–29.99 kg/m^2^).	Twelve weeks of walking 5000 steps/day. Diet (1000–1500 kcal/day).	Ghrelin levels significantly decreased.
Long-term training (≥12 weeks) with higher plasma ghrelin levels			
Leidy et al. [142]	Fifteen females (age 20.2 ± 1.4 years; BMI, range 18–25 kg/m^2^).	Aerobic exercise at 70–80% of HR_max_ for 12 weeks. Diet.	Ghrelin increased from 2593 ± 995 to 4449 ± 2234 pg/mL, +71.6% (*p* < 0.05). The %body fat decreased from 30.5 ± 3.5% (*p* < 0.05).
FosterSchubert et al. [143]	Eighty-seven postmenopausal women (age 60.7 ± 6.75 years; BMI, 30.4 ± 4.1 kg/m^2^).	Aerobic training, cycling at 60–75% of HR_max_, 45 min/day for 12 weeks.	Ghrelin increased by +24.1% (*p* < 0.05). BM decreased (*p* < 0.05).
Mizia-Stec et al. [144]	Thirty-seven obese premenopausal females (age 29 ± 52 years; BMI, 36.5 ± 5 kg/m^2^).	Twelve weeks of aerobic exercise x 60 min at 65% of HR_max_. Diet (1000 kcal/day).	Ghrelin increased from 66.9 ± 13.7 to 73.9 ± 15.4 pg/mL, +10.5% (*p* = 0.005). BMI decreased from 36.5 ± 5.4 to 33.4 ± 5.2 kg/m^2^ (*p* = 0.001).
Kelishadi et al. [145]	Ninety-two female and male obese children (age 7.7 ± 1.2 years; BMI, 21.1 ± 2.5 kg/m^2^).	Twenty-four weeks, 40 min training. Diet.	Ghrelin decreased (*p* < 0.05). BMI decreased (*p* < 0.05).
Konopko-Zubrzycka et al. [146]	Twenty-one obese females and males (age 41 ± 11.9 years; BMI, 47.3 ± 5.7 kg/m^2^).	Twenty-four weeks of walking for 45 min. Bioenterics intragastric balloon diet (1500 kcal/d).	Ghrelin increased from 621.9 ± 182.4 to 903.9 ± 237 pg/mL, +43.3% (*p* < 0.01), and gradually returned to baseline 3 months post-balloon removal. BM decreased.
Gueugnon et al. [147]	Thirty-two female and male obese adolescents (age 14.3 ± 0.3 years; BMI, 35.6 ± 0.7 kg/m^2^).	Twenty-eight weeks of aerobic training, 45–60 min at 50–85% of MAP. Diet (2300–2500 kcal/day).	Ghrelin increased (*p* < 0.05). BMI decreased (*p* < 0.001).
Kadoglou et al. [148]	Fifty-four overweight females and males with type 2 diabetes mellitus diagnosis (age range, 50–70 years; BMI, 32.1 ± 3.77 kg/m^2^).	Twelve weeks of aerobic training for 45–60 min/day at 60–75% of HR_max._	Ghrelin increased from 2140 ± 710 to 3870 ± 1070 pg/mL, +80.8% (*p* > 0.05). BMI did not change from 32.1 ± 3.77 to 31.98 ± 3.03 kg/m^2^ (*p* > 0.05).
Markofski et al. [149]	Twenty-nine older, healthy females and males (age 71.2 ± 5 years).	Twelve weeks of aerobic and resistance training for 20 min/day at 60–70% of HRr and 80% of 1 RM.	Ghrelin increased from 32.9 ± 4.0 to 48.2 ± 6.0 pg/mL, +47% (*p* < 0.01). BMI did not change (*p* > 0.05).
Kim et al. [150]	Eighteen untrained healthy males (age, 23.6 ± 2.8 years). Group 1: BMI, 23.6 ± 2.8 kg/m^2^, isocaloric high protein diet. Group 2: 24.5 ± 2.8 kg/m^2^, standard diet.	Twelve weeks of resistance training for 50–80 min/day at 60–80% 1 RM.	Group 1: Ghrelin increased from 658.2 ± 73.8 to 817.2 ± 87.0 pg/mL, +25% (*p* = 0.001). The %body fat decreased from 22.4 ± 5.8% to 20.0 ± 4.9% (*p* < 0.05). Group 2: Ghrelin did not change from 707 ± 133 to 760 ± 91.2 pg/mL, +9.3% (*p* > 0.05). The %body fat did not change from 19.2 ± 7.9% to 18.3 ± 7.3% (*p* > 0.05).
Kang et al. [151]	Twenty-six obese females. Intervention group (age, 50.1 ± 3.8 years; BMI, 31.8 ± 3.2 kg/m^2^). Control group (age, 49.84 ± 2.96 years; BMI, 30.4 ± 2.3 kg/m^2^).	Twelve weeks of aerobic and resistance training for 50 min at 12–14 of RPE.	Ghrelin increased from 588.5 ± 139 pg/mL to 821.4 ± 197.0, +39.5% (*p* < 0.001). The %body fat decreased from 38.7 ± 3.2% to 36.9 ± 3.5% (*p* < 0.05).
Tremblay et al. [29]	One hundred overweight adults/elderly females and males with metabolic syndrome group (age range 50–70 years). Group 1: BM, 85.4 ± 12.4 kg. Group 2: BM, 94.0 ± 13.7 kg. Group 3: BM, 89.0 ± 12.7 kg.	Twenty-four weeks of 90 min/day of endurance plus 90 min/week of resistance training. Group 1: High resistance (at 65–85% of 10 RM), moderate endurance (30% of VO_2peak_) training. Group 2: Moderate resistance (at 30% of 10 RM), high endurance (70% of VO_2peak_ training). Group 3: Moderate resistance (at 30% of 10 RM) endurance, for resistance (30% of VO_2peak_).	Ghrelin significantly increased after day 21 (*p* < 0.001), returning to baseline levels between months 6 and 12 as body weight and fat plateaued. Group 1: Body fat decreased from 27.7 ± 7.6 to 22.1 ± 6.9 kg (*p* < 0.05). Group 2: Body fat decreased from 29.6 ± 0.7 to 26.3 ± 6.8 kg (*p* < 0.05). Group 3: Body fat decreased from 32.3 ± 7.5 to 28.3 ± 6.8 kg (*p* < 0.05).
Ataeinosrat et al. [152]	Forty-four obese males (age 27.5 ± 9.4 years; BMI, 32.9 ± 1.2 kg/m^2^).	Twelve weeks of 70 min at 50% of 1 RM training. Group 1: Circuit resistance training. Group 2: Traditional resistance. Group 3: Interval resistance training.	Group 1: Ghrelin increased from 650 ± 14 to 870 ± 11 pg/mL, +33.8% (*p* < 0.05). Body fat decreased from 29.9 ± 1.1 to 27.2 ± 0.8 kg (*p* < 0.05). Group 2: Ghrelin increased from 659 ± 20 to 779 ± 17 pg/mL, +18.2% (*p* < 0.05). Body fat did not change from 29.9 ± 1.1 to 28.5 ± 0.8 kg (*p* > 0.05). Group 3: Ghrelin increased from 669 ± 31 to 866 ± 18 pg/mL, +29.4% (*p* < 0.05). Body fat decreased from 30.4 ± 1.0 to 26.8 ± 1.0 kg (*p* < 0.05).
Najafi et al. [12]	Forty-five females with precocious puberty (age range 6–8 years). Group 1: Medication plus training, BMI, 17.9 ± 0.6 kg/m^2^. Group 2: Medication, BMI, 17.8 ± 0.68 kg/m^2^. Group 3: Control, BMI, 16.5 ± 0.49 kg/m^2^.	Twelve weeks of aerobic training, 20–75 min/day at 45–75% of HR_max_.	Group 1: Ghrelin increased from 8210 ± 1400 to 9110 ± 1350 pg/mL, +11% (*p* = 0.001). BMI decreased from 17.9 ± 0.6 to 17.1 ± 0.44 kg/m^2^ (*p* < 0.05). Group 2: Ghrelin did not change from 8340 ± 1210 to 8400 ± 1210 pg/mL, +0.7% (*p* > 0.05). BMI did not change from 17.8 ± 0.68 to 17.9 ± 0.72 kg/m^2^ (*p* > 0.05). Group 3: Ghrelin did not change from 9880 ± 1300 to 9900 ± 1800 pg/mL, +0.2% (*p* > 0.05). BMI did not change from 16.5 ± 0.49 to 16.6 ± 0.29 kg/m^2^ (*p* > 0.05).
Very long-term training (≥48 weeks) with stable plasma ghrelin levels			
Campos et al. [153]	Forty-two female and male obese adolescents (age 16.22 ± 1.35 years). Group1: BMI, 35.82 ± 4.52 kg/m^2^. Group 2: BMI, 37.6 ± 5.44 kg/m^2^.	Forty-eight weeks of 60 min/day. Group 1: Aerobic training. Group 2: Aerobic plus resistance (6–12 of RM) training.	Group 1: Ghrelin did not change from 7660 ± 6230 to 8890 ± 6450 pg/mL, +16.1% (*p* > 0.05). BMI decreased from 35.82 ± 4.52 to 32.06 ± 4.92 kg/m^2^ (*p* < 0.05). The %body fat decreased from 43.97 ± 6.46 to 38.44 ± 8.97% (*p* < 0.05); Group 2: Ghrelin increased from 1240 ± 480 to 1470 ± 1700 pg/mL, +18.6% (*p* < 0.05). BMI decreased from 37.6 ± 5.44 to 32.6 ± 4.56 kg/m^2^ (*p* < 0.05). The %body fat decreased from 50.5 ± 6.46 to 45.2 ± 8.14% (*p* < 0.01).
Very long-term training (≥48 weeks) with higher plasma ghrelin levels			
Foster-Schubert et al. [143]	Eighty-seven postmenopausal obese women (age 60.7 ± 6.75 years; BM, 81 ± 14.1 Kg; BMI, 30.4 ± 4.1 kg/m^2^).	Forty-eight weeks of aerobic training, for 45 min/day at 60–75% of HR_max_. Diet (1200–2000 kcal/day).	Ghrelin increased by 32 ± 16 pg/mL (*p* < 0.05). BM decreased from 81.7 ± 1.4 kg to 80.5 ± 0.3 (*p* < 0.05).
Mason et al. [154]	Three hundred ninety-nine overweight/ obese postmenopausal women (age 57.9 ± 5 years; BMI, 30.9 ± 4.0 kg/m^2^).	Twelve months of moderate-to-vigorous intensity exercise for 45 min/day (5 days/week) at 70–85% of HR_max_. Diet: 1200–2000 kcal/day.	Ghrelin significantly increased in the diet plus exercise (+7.4%, *p* = 0.008) group but not in either the diet (+6.5%, *p* = 0.07) or exercise (+1.0%, *p* = 0.53) groups. BM decreased by 2.4% (*p* = 0.03) in the exercise group, by 8.5% (*p* < 0.001) in the diet group, and by 10.8% (*p* < 0.001) in the diet plus exercise group.

Data are expressed as mean ± SD. HRmax, maximum heart rate; HRr, heart rate reserve; BF, body fat; BM, body mass; BMI, body mass index; MAP, maximal aerobic power; RM, repetition maximum; RPE, rate of perceived exertion; VO_2max_, maximal oxygen uptake; VO_2peak_, peak of oxygen consumption.

## Supplementary Materials

The supporting information can be downloaded at: https://www.mdpi.com/article/10.3390/ijms26104753/s1.

## References

[1] Cifuentes L., Acosta A. (2022). Homeostatic regulation of food intake. Clin. Res. Hepatol. Gastroenterol..

[2] Prinz P., Stengel A. (2017). Control of Food Intake by Gastrointestinal Peptides: Mechanisms of Action and Possible Modulation in the Treatment of Obesity. J. Neurogastroenterol. Motil..

[3] Suzuki K., Jayasena C.N., Bloom S.R. (2012). Obesity and appetite control. Exp. Diabetes Res..

[4] Kojima M., Hosoda H., Date Y., Nakazato M., Matsuo H., Kangawa K. (1999). Ghrelin is a growth-hormone-releasing acylated peptide from stomach. Nature.

[5] Diz-Chaves Y. (2011). Ghrelin, appetite regulation, and food reward: Interaction with chronic stress. Int. J. Pept..

[6] Pradhan G., Samson S.L., Sun Y. (2013). Ghrelin: Much more than a hunger hormone. Curr. Opin. Clin. Nutr. Metab. Care.

[7] Williams D.L., Cummings D.E. (2005). Regulation of ghrelin in physiologic and pathophysiologic states. J. Nutr..

[8] Khatib N., Gaidhane S., Gaidhane A.M., Khatib M., Simkhada P., Gode D., Zahiruddin Q.S. (2014). Ghrelin: Ghrelin as a regulatory Peptide in growth hormone secretion. J. Clin. Diagn. Res..

[9] Kraemer W.J., Ratamess N.A. (2004). Fundamentals of resistance training: Progression and exercise prescription. Med. Sci. Sports Exerc..

[10] Alyar G., Umudum F.Z., Akbaş N. (2024). Changes in ghrelin, GLP-1, and PYY levels after diet and exercise in obese individuals. Rev. Assoc. Med. Bras..

[11] Liu H.W., Cheng H.C., Tsai S.H., Shao Y.T. (2023). Effects of acute resistance exercise with different loads on appetite, appetite hormones and autonomic nervous system responses in healthy young men. Appetite.

[12] Najafi R., Heidarianpour A., Shokri E., Shokri B. (2023). Ameliorative effects of aerobic training in girls with precocious puberty: Role of leptin and ghrelin. Sci. Rep..

[13] Ouerghi N., Feki M., Bragazzi N.L., Knechtle B., Hill L., Nikolaidis P.T., Bouassida A. (2021). Ghrelin Response to Acute and Chronic Exercise: Insights and Implications from a Systematic Review of the Literature. Sports Med..

[14] Carreira M.C., Crujeiras A.B., Andrade S., Monteiro M.P., Casanueva F.F. (2013). Ghrelin as a GH-releasing factor. Endocr. Dev..

[15] Abassi W., Ouerghi N., Ghouili H., Haouami S., Bouassida A. (2020). Greater effects of high- compared with moderate-intensity interval training on thyroid hormones in overweight/obese adolescent girls. Horm. Mol. Biol. Clin. Investig..

[16] Abassi W., Ouerghi N., Nikolaidis P.T., Hill L., Racil G., Knechtle B., Feki M., Bouassida A. (2022). Interval Training with Different Intensities in Overweight/Obese Adolescent Females. Int. J. Sports Med..

[17] Abassi W., Ouerghi N., Feki M., Jebabli N., Andrade M.S., Bouassida A., Sousa C.V., Nikolaidis P.T., Weiss K., Knechtle B. (2023). Effects of moderate-vs. high-intensity interval training on physical fitness, enjoyment, and affective valence in overweight/obese female adolescents: A pre-/post-test study. Eur. Rev. Med. Pharmacol. Sci..

[18] Abassi W., Ouerghi N., Hammami M.B., Jebabli N., Feki M., Bouassida A., Weiss K., Knechtle B. (2025). High-Intensity Interval Training Reduces Liver Enzyme Levels and Improves MASLD-Related Biomarkers in Overweight/Obese Girls. Nutrients.

[19] Ouerghi N., Brini S., Zaouali M., Feki M., Tabka Z., Bouassida A. (2019). Ghrelin is not altered after acute exercises at different intensities in overweight middle-aged individuals. Sci. Sports.

[20] Russel R.D., Willis K.S., Ravussin E., Larson-Meyer E.D. (2009). Effects of endurance running and dietary fat on circulating ghrelin and peptide YY. J. Sports Sci. Med..

[21] Jürimäe J., Rämson R., Mäestu J., Purge P., Jürimäe T., Arciero P.J., von Duvillard S.P. (2009). Plasma visfatin and ghrelin response to prolonged sculling in competitive male rowers. Med. Sci. Sports Exerc..

[22] Li S., Guo R., Wang J., Zheng X., Zhao S., Zhang Z., Yu W., Li S., Zheng P. (2023). The effect of blood flow restriction exercise on N-lactoylphenylalanine and appetite regulation in obese adults: A cross-design study. Front. Endocrinol..

[23] Tobin S.Y., Cornier M.A., White M.H., Hild A.K., Simonsen S.E., Melanson E.L., Halliday T.M. (2021). The effects of acute exercise on appetite and energy intake in men and women. Physiol. Behav..

[24] Laursen T.L., Zak R.B., Shute R.J., Heesch M.W.S., Dinan N.E., Bubak M.P., La Salle D.T., Slivka D.R. (2017). Leptin, adiponectin, and ghrelin responses to endurance exercise in different ambient conditions. Temperature.

[25] Ahmadi S.M., Fathi M., RashidLamir A., Aminian F. (2019). Effects of 8 Weeks Aerobic Training on Plasma Ghrelin Level and Ghrelin Lymphocyte Gene Expression in Elderly Men. Salmand Iran. J. Ageing.

[26] Fico B.G., Alkatan M., Tanaka H. (2020). No changes in appetite-related hormones following swimming and cycling exercise interventions in adults with obesity. Int. J. Exerc. Sci..

[27] Cho G.J., Han S.W., Shin J.H., Kim T. (2017). Effects of intensive training on menstrual function and certain serum hormones and peptides related to the female reproductive system. Medicine.

[28] Liao J., Huang J., Wang S., Xiang M., Wang D., Deng H., Yin H., Xu F., Hu M. (2021). Effects of exercise and diet intervention on appetite-regulating hormones associated with miRNAs in obese children. Eat. Weight Disord. Stud. Anorexia Bulim. Obes..

[29] Tremblay A., Dutheil F., Drapeau V., Metz L., Lesour B., Chapier R., Pereira B., Verney J., Baker J.S., Vinet A. (2019). Long-term effects of high-intensity resistance and endurance exercise on plasma leptin and ghrelin in overweight individuals: The RESOLVE Study. Appl. Physiol. Nutr. Metab..

[30] Ibrahim Abdalla M.M. (2015). Ghrelin—Physiological Functions and Regulation. Eur. Endocrinol..

[31] Müller T.D., Nogueiras R., Andermann M.L., Andrews Z.B., Anker S.D., Argente J., Batterham R.L., Benoit S.C., Bowers C.Y., Broglio F. (2015). Ghrelin. Mol. Metab..

[32] Zhu X., Cao Y., Voogd K., Steiner D.F. (2007). On the processing of proghrelin to ghrelin. J. Biol. Chem..

[33] Kojima M., Kangawa K. (2005). Ghrelin: Structure and function. Physiol. Rev..

[34] Nunez-Salces M., Li H., Feinle-Bisset C., Young R.L., Page A.J. (2021). The regulation of gastric ghrelin secretion. Acta Physiol..

[35] Sato T., Nakamura Y., Shiimura Y., Ohgusu H., Kangawa K., Kojima M. (2012). Structure, regulation and function of ghrelin. J. Biochem..

[36] Hartman M.L., Faria A.C., Vance M.L., Johnson M.L., Thorner M.O., Veldhuis J.D. (1991). Temporal structure of in vivo growth hormone secretory events in humans. Am. J. Physiol.-Regul. Integr. Comp. Physiol..

[37] Thorner M.O., Chapman I.M., Gaylinn B.D., Pezzoli S.S., Hartman M.L. (1997). Growth hormone-releasing hormone and growth hormone-releasing peptide as therapeutic agents to enhance growth hormone secretion in disease and aging. Recent Progress in Hormone Research.

[38] Lanfranco F., Motta G., Baldi M., Gasco V., Grottoli S., Benso A., Broglio F., Ghigo E. (2010). Ghrelin and anterior pituitary function. Front. Horm. Res..

[39] Perchard R., Clayton P.E. (2017). Ghrelin and Growth. Endocr. Dev..

[40] Druce M.R., Wren A.M., Park A.J., Milton J.E., Patterson M., Frost G., Ghatei M.A., Small C., Bloom S.R. (2005). Ghrelin increases food intake in obese as well as lean subjects. Int. J. Obes..

[41] Cummings D.E., Purnell J.Q., Frayo R.S., Schmidova K., Wisse B.E., Weigle D.S. (2001). A preprandial rise in plasma ghrelin levels suggests a role in meal initiation in humans. Diabetes.

[42] Natalucci G., Riedl S., Gleiss A., Zidek T., Frisch H. (2005). Spontaneous 24-h ghrelin secretion pattern in fasting subjects: Maintenance of a meal-related pattern. Eur. J. Endocrinol..

[43] Tschöp M., Wawarta R., Riepl R.L., Friedrich S., Bidlingmaier M., Landgraf R., Folwaczny C. (2001). Post-prandial decrease of circulating human ghrelin levels. J. Endocrinol. Investig..

[44] Chen C.Y., Asakawa A., Fujimiya M., Lee S.D., Inui A. (2009). Ghrelin gene products and the regulation of food intake and gut motility. Pharmacol. Rev..

[45] Lee H.M., Wang G., Englander E.W., Kojima M., Greeley G.H. (2002). Ghrelin, a new gastrointestinal endocrine peptide that stimulates insulin secretion: Enteric distribution, ontogeny, influence of endocrine, and dietary manipulations. Endocrinology.

[46] Reimer M.K., Pacini G., Ahrén B. (2003). Dose-dependent inhibition by ghrelin of insulin secretion in the mouse. Endocrinology.

[47] Tong J., Prigeon R.L., Davis H.W., Bidlingmaier M., Kahn S.E., Cummings D.E., Tschöp M.H., D’Alessio D. (2010). Ghrelin suppresses glucose-stimulated insulin secretion and deteriorates glucose tolerance in healthy humans. Diabetes.

[48] Andralojc K.M., Mercalli A., Nowak K.W., Albarello L., Calcagno R., Luzi L., Bonifacio E., Doglioni C., Piemonti L. (2009). Ghrelin-producing epsilon cells in the developing and adult human pancreas. Diabetologia.

[49] Benitez C.M., Goodyer W.R., Kim S.K. (2012). Deconstructing pancreas developmental biology. Cold Spring Harb. Perspect. Biol..

[50] Poher A.L., Tschöp M.H., Müller T.D. (2018). Ghrelin regulation of glucose metabolism. Peptides.

[51] Qader S.S., Lundquist I., Ekelund M., Håkanson R., Salehi A. (2005). Ghrelin activates neuronal constitutive nitric oxide synthase in pancreatic islet cells while inhibiting insulin release and stimulating glucagon release. Regul. Pept..

[52] Wiedemann T., Bielohuby M., Müller T.D., Bidlingmaier M., Pellegata N.S. (2016). Obesity in MENX rats is accompanied by high circulating levels of ghrelin and improved insulin sensitivity. Diabetes.

[53] Chuang J.C., Sakata I., Kohno D., Perello M., Osborne-Lawrence S., Repa J.J., Zigman J.M. (2011). Ghrelin directly stimulates glucagon secretion from pancreatic alpha-cells. Mol. Endocrinol..

[54] Yi C.X., Heppner K.M., Kirchner H., Tong J., Bielohuby M., Gaylinn B.D., Müller T.D., Bartley E., Davis H.W., Zhao Y. (2012). The GOAT-ghrelin system is not essential for hypoglycemia prevention during prolonged calorie restriction. PLoS ONE.

[55] Park S., Jiang H., Zhang H., Smith R.G. (2012). Modification of ghrelin receptor signaling by somatostatin receptor-5 regulates insulin release. Proc. Natl. Acad. Sci. USA.

[56] Soriano-Guillén L., Barrios V., Martos G., Chowen J.A., Campos-Barros A., Argente J. (2004). Effect of oral glucose administration on ghrelin levels in obese children. Eur. J. Endocrinol..

[57] Martos-Moreno G.A., Barrios V., Soriano-Guillén L., Argente J. (2006). Relationship between adiponectin levels, acylated ghrelin levels, and short-term body mass index changes in children with diabetes mellitus type 1 at diagnosis and after insulin therapy. Eur. J. Endocrinol..

[58] Cowley M.A., Smith R.G., Diano S., Tschöp M., Pronchuk N., Grove K.L., Strasburger C.J., Bidlingmaier M., Esterman M., Heiman M.L. (2003). The distribution and mechanism of action of ghrelin in the CNS demonstrates a novel hypothalamic circuit regulating energy homeostasis. Neuron.

[59] Kamegai J., Tamura H., Shimizu T., Ishii S., Sugihara H., Wakabayashi I. (2001). Chronic central infusion of ghrelin increases hypothalamic neuropeptide Y and Agouti-related protein mRNA levels and body weight in rats. Diabetes.

[60] Notaro N.M., Dyck D.J. (2024). Regulation of peripheral tissue substrate metabolism by the gut-derived hormone ghrelin. Metab. Open.

[61] Theander-Carrillo C., Wiedmer P., Cettour-Rose P., Nogueiras R., Perez-Tilve D., Pfluger P., Castaneda T.R., Muzzin P., Schürmann A., Szanto I. (2006). Ghrelin action in the brain controls adipocyte metabolism. J. Clin. Investig..

[62] Tschöp M., Smiley D.L., Heiman M.L. (2000). Ghrelin Induces Adiposity in Rodents. Nature.

[63] Hucik B., Lovell A.J., Hoecht E.M., Cervone D.T., Mutch D.M., Dyck D.J. (2021). Regulation of adipose tissue lipolysis by ghrelin is impaired with high-fat diet feeding and is not restored with exercise. Adipocyte.

[64] Wells T. (2009). Ghrelin—Defender of fat. Prog. Lipid Res..

[65] Davies J.S., Kotokorpi P., Eccles S.R., Barnes S.K., Tokarczuk P.F., Allen S.K., Whitworth H.S., Guschina I.A., Evans B.A., Mode A. (2009). Ghrelin induces abdominal obesity via GHS-R-dependent lipid retention. Mol. Endocrinol..

[66] Wren A.M., Small C.J., Abbott C.R., Dhillo W.S., Seal L.J., Cohen M.A., Batterham R.L., Taheri S., Stanley S.A., Ghatei M.A. (2001). Ghrelin causes hyperphagia and obesity in rats. Diabetes.

[67] Li Z., Xu G., Qin Y., Zhang C., Tang H., Yin Y., Xiang X., Li Y., Zhao J., Mulholland M. (2014). Ghrelin promotes hepatic lipogenesis by activation of mTOR-PPARγ signaling pathway. Proc. Natl. Acad. Sci. USA.

[68] Ezquerro S., Méndez-Giménez L., Becerril S., Moncada R., Valentí V., Catalán V., Gómez-Ambrosi J., Frühbeck G., Rodríguez A. (2016). Acylated and desacyl ghrelin are associated with hepatic lipogenesis, β-oxidation and autophagy: Role in NAFLD amelioration after sleeve gastrectomy in obese rats. Sci. Rep..

[69] Anderson K.C., Mardian T., Stephenson B., Grammer E.E., Stahl M.E., Weeldreyer N.R., Liu Z., Love K.M., Kranz S., Allen J.D. (2024). The Impact of Exercise Intensity and Sex on Endogenous Ghrelin Levels and Appetite in Healthy Humans. J. Endocr. Soc..

[70] Beauloye V., Diene G., Kuppens R., Zech F., Winandy C., Molinas C., Faye S., Kieffer I., Beckers D., Nergårdh R. (2016). High unacylated ghrelin levels support the concept of anorexia in infants with Prader-Willi syndrome. Orphanet J. Rare Dis..

[71] Porporato P.E., Filigheddu N., Reano S., Ferrara M., Angelino E., Gnocchi V.F., Prodam F., Ronchi G., Fagoonee S., Fornaro M. (2013). Acylated and unacylated ghrelin impair skeletal muscle atrophy in mice. J. Clin. Investig..

[72] Balasubramaniam A., Joshi R., Su C., Friend L.A., Sheriff S., Kagan R.J., James J.H. (2009). Ghrelin inhibits skeletal muscle protein breakdown in rats with thermal injury through normalizing elevated expression of E3 ubiquitin ligases MuRF1 and MAFbx. Am. J. Physiol. Regul. Integr. Comp. Physiol..

[73] Deboer M.D., Zhu X., Levasseur P.R., Inui A., Hu Z., Han G., Mitch W.E., Taylor J.E., Halem H.A., Dong J. (2008). Ghrelin treatment of chronic kidney disease: Improvements in lean body mass and cytokine profile. Endocrinology.

[74] Sugiyama M., Yamaki A., Furuya M., Inomata N., Minamitake Y., Ohsuye K., Kangawa K. (2012). Ghrelin improves body weight loss and skeletal muscle catabolism associated with angiotensin II-induced cachexia in mice. Regul. Pept..

[75] Filigheddu N., Gnocchi V.F., Coscia M., Cappelli M., Porporato P.E., Taulli R., Traini S., Baldanzi G., Chianale F., Cutrupi S. (2007). Ghrelin and des-acyl ghrelin promote differentiation and fusion of C2C12 skeletal muscle cells. Mol. Biol. Cell.

[76] Fukushima N., Hanada R., Teranishi H., Fukue Y., Tachibana T., Ishikawa H., Takeda S., Takeuchi Y., Fukumoto S., Kangawa K. (2005). Ghrelin directly regulates bone formation. J. Bone Miner. Res..

[77] Nikolopoulos D., Theocharis S., Kouraklis G. (2010). Ghrelin, another factor affecting bone metabolism. Med. Sci. Monit..

[78] van der Velde M., Delhanty P., van der Eerden B., van der Lely A.J., van Leeuwen J. (2008). Ghrelin and bone. Vitam. Horm..

[79] Costa J.L., Naot D., Lin J.M., Watson M., Callon K.E., Reid I.R., Grey A.B., Cornish J. (2011). Ghrelin is an Osteoblast Mitogen and Increases Osteoclastic Bone Resorption In Vitro. Int. J. Pept..

[80] Roelen B.A., Dijke P.T. (2003). Controlling mesenchymal stem cell differentiation by TGFBeta family members. J. Orthop. Sci..

[81] Wells T., Houston P.A. (2001). Skeletal growth acceleration with growth hormone secretagogues in transgenic growth retarded rats: Pattern-dependent effects and mechanisms of desensitization. J. Neuroendocrinol..

[82] Monson J.P., Drake W.M., Carroll P.V., Weaver J.U., Rodriguez-Arnao J., Savage M.O. (2002). Influence of growth hormone on accretion of bone mass. Horm. Res..

[83] Delhanty P.J., van der Eerden B.C., van der Velde M., Gauna C., Pols H.A., Jahr H., Chiba H., van der Lely A.J., van Leeuwen J.P. (2006). Ghrelin and unacylated ghrelin stimulate human osteoblast growth via mitogen-activated protein kinase (MAPK)/phosphoinositide 3-kinase (PI3K) pathways in the absence of GHS-R1a. J. Endocrinol..

[84] Kim S.W., Her S.J., Park S.J., Kim D., Park K.S., Lee H.K., Han B.H., Kim M.S., Shin C.S., Kim S.Y. (2005). Ghrelin stimulates proliferation and differentiation and inhibits apoptosis in osteoblastic MC3T3-E1 cells. Bone.

[85] Maccarinelli G., Sibilia V., Torsello A., Raimondo F., Pitto M., Giustina A., Netti C., Cocchi D. (2005). Ghrelin regulates proliferation and differentiation of osteoblastic cells. J. Endocrinol..

[86] Barre R., Beton N., Batut A., Accabled F., Sales de Gauzy J., Auriol F., Eddiry S., Tauber M., Laurencin S., Salles J.P. (2020). Ghrelin uses the GHS-R1a/Gi/cAMP pathway and induces differentiation only in mature osteoblasts. This ghrelin pathway is impaired in AIS patients. Biochem. Biophys. Rep..

[87] Napoli N., Pedone C., Pozzilli P., Lauretani F., Bandinelli S., Ferrucci L., Incalzi R.A. (2011). Effect of ghrelin on bone mass density: The InChianti study. Bone.

[88] Makovey J., Chen J.S., Hayward C., Williams F.M., Sambrook P.N. (2009). Association between serum cholesterol and bone mineral density. Bone.

[89] Pérez-Castrillón J.L., Justo I., Sanz A., San Miguel A., Mazón M.A., Abad L., Vega G., Dueñas A. (2007). Ghrelin and bone mass in postmenopausal hypertensive women. Ann. Nutr. Metab..

[90] Akalu Y., Molla M.D., Dessie G., Ayelign B. (2020). Physiological Effect of Ghrelin on Body Systems. Int. J. Endocrinol..

[91] Nagaya N., Uematsu M., Kojima M., Ikeda Y., Yoshihara F., Shimizu W., Hosoda H., Hirota Y., Ishida H., Mori H. (2001). Chronic administration of ghrelin improves left ventricular dysfunction and attenuates development of cardiac cachexia in rats with heart failure. Circulation.

[92] Nagaya N., Moriya J., Yasumura Y., Uematsu M., Ono F., Shimizu W., Ueno K., Kitakaze M., Miyatake K., Kangawa K. (2004). Effects of ghrelin administration on left ventricular function, exercise capacity, and muscle wasting in patients with chronic heart failure. Circulation.

[93] Leite-Moreira A.F., Rocha-Sousa A., Henriques-Coelho T. (2008). Cardiac, skeletal, and smooth muscle regulation by ghrelin. Vitam. Horm..

[94] Soeki T., Koshiba K., Niki T., Kusunose K., Yamaguchi K., Yamada H., Wakatsuki T., Shimabukuro M., Minakuchi K., Kishimoto I. (2014). Effect of ghrelin on autonomic activity in healthy volunteers. Peptides.

[95] Lilleness B.M., Frishman W.H. (2016). Ghrelin and the Cardiovascular System. Cardiol. Rev..

[96] Okumura H., Nagaya N., Enomoto M., Nakagawa E., Oya H., Kangawa K. (2002). Vasodilatory effect of ghrelin, an endogenous peptide from the stomach. J. Cardiovasc. Pharmacol..

[97] Fang W., Zhang M., Qu X., Yuan F., Yang Y., Xu L., Dai J., Wang W., Fei J., Hou X. (2015). Ghrelin receptor deficiency aggravates atherosclerotic plaque instability and vascular inflammation. Front. Biosci..

[98] Ukkola O. (2015). Ghrelin and atherosclerosis. Curr. Opin. Lipidol..

[99] Li W.G., Gavrila D., Liu X., Wang L., Gunnlaugsson S., Stoll L.L., McCormick M.L., Sigmund C.D., Tang C., Weintraub N.L. (2004). Ghrelin inhibits proinflammatory responses and nuclear factor-kappaB activation in human endothelial cells. Circulation.

[100] Waseem T., Duxbury M., Ito H., Ashley S.W., Robinson M.K. (2008). Exogenous ghrelin modulates release of pro-inflammatory and anti-inflammator cytokines in LPS-stimulated macrophages through distinct signaling pathways. Surgery.

[101] Takata A., Takiguchi S., Miyazaki Y., Miyata H., Takahashi T., Kurokawa Y., Yamasaki M., Nakajima K., Mori M., Kangawa K. (2015). Randomized Phase II Study of the Anti-inflammatory Effect of Ghrelin During the Postoperative Period of Esophagectomy. Ann. Surg..

[102] Farokhnia M., Portelli J., Lee M.R., McDiarmid G.R., Munjal V., Abshire K.M., Battista J.T., Browning B.D., Deschaine S.L., Akhlaghi F. (2020). Effects of exogenous ghrelin administration and ghrelin receptor blockade, in combination with alcohol, on peripheral inflammatory markers in heavy-drinking individuals: Results from two human laboratory studies. Brain Res..

[103] Ma Y., Zhang H., Guo W., Yu L. (2022). Potential role of ghrelin in the regulation of inflammation. FASEB J..

[104] Lee J., Lim E., Kim Y., Li E., Park S. (2010). Ghrelin attenuates kainic acid-induced neuronal cell death in the mouse hippocampus. J. Endocrinol..

[105] Zhang H., Cui Z., Luo G., Zhang J., Ma T., Hu N., Cui T. (2013). Ghrelin attenuates intestinal ischemia/reperfusion injury in mice by activating the mTOR signaling pathway. Int. J. Mol. Med..

[106] El-Shaer N.O., El Gazzar W.B., Allam M.M., Anwer H.M. (2021). Ghrelin ameliorated inflammation and oxidative stress in isoproterenol induced myocardial infarction through the endothelial nitric oxide synthase (eNOS)/nuclear factor erythroid 2-related factor-2 (NRF2)/heme oxygenase-1 (HO-1) signaling pathway. J. Physiol. Pharmacol..

[107] Liberati A., Altman D.G., Tetzlaff J., Mulrow C., Gøtzsche P.C., Ioannidis J.P., Clarke M., Devereaux P.J., Kleijnen J., Moher D. (2009). The PRISMA statement for reporting systematic reviews and meta-analyses of studies that evaluate health care interventions: Explanation and elaboration. J. Clin. Epidemiol..

[108] Dall R., Kanaley J., Hansen T.K., Møller N., Christiansen J.S., Hosoda H., Kangawa K., Jørgensen J.O. (2002). Plasma ghrelin levels during exercise in healthy subjects and in growth hormone-deficient patients. Eur. J. Endocrinol..

[109] Kraemer R.R., Durand R.J., Acevedo E.O., Johnson L.G., Kraemer G.R., Hebert E.P., Castracane V.D. (2004). Rigorous running increases growth hormone and insulin-like growth factor-I without altering ghrelin. Exp. Biol. Med..

[110] Schmidt A., Maier C., Schaller G., Nowotny P., Bayerle-Eder M., Buranyi B., Luger A., Wolzt M. (2004). Acute exercise has no effect on ghrelin plasma concentrations. Horm. Metab. Res..

[111] Zoladz J.A., Konturek S.J., Duda K., Majerczak J., Sliwowski Z., Grandys M., Bielanski W. (2005). Effect of moderate incremental exercise, performed in fed and fasted state on cardio-respiratory variables and leptin and ghrelin concentrations in young healthy men. J. Physiol. Pharmacol..

[112] Erdmann J., Tahbaz R., Lippl F., Wagenpfeil S., Schusdziarra V. (2007). Plasma ghrelin levels during exercise—Effects of intensity and duration. Regul. Pept..

[113] Jürimäe J., Hofmann P., Jürimäe T., Palm R., Mäestu J., Purge P., Sudi K., Rom K., von Duvillard S.P. (2007). Plasma ghrelin responses to acute sculling exercises in elite male rowers. Eur. J. Appl. Physiol..

[114] Marzullo P., Salvadori A., Brunani A., Verti B., Walker G.E., Fanari P., Tovaglieri I., De Medici C., Savia G., Liuzzi A. (2008). Acylated ghrelin decreases during acute exercise in the lean and obese state. Clin. Endocrinol..

[115] Thomas G.A., Kraemer W.J., Comstock B.A., Dunn-Lewis C., Volek J.S., Denegar C.R., Maresh C.M. (2012). Effects of resistance exercise and obesity level on ghrelin and cortisol in men. Metabolism.

[116] Crabtree D.R., Blannin A.K. (2015). Effects of exercise in the cold on Ghrelin, PYY, and food intake in overweight adults. Med. Sci. Sports Exerc..

[117] Toshinai K., Kawagoe T., Shimbara T., Tobina T., Nishida Y., Mondal M.S., Yamaguchi H., Date Y., Tanaka H., Nakazato M. (2007). Acute incremental exercise decreases plasma ghrelin level in healthy men. Horm. Metab. Res..

[118] Malkova D., McLaughlin R., Manthou E., Wallace A.M., Nimmo M.A. (2008). Effect of moderate-intensity exercise session on preprandial and postprandial responses of circulating ghrelin and appetite. Horm. Metab. Res..

[119] Stokes K.A., Sykes D., Gilbert K.L., Chen J.W., Frystyk J. (2010). Brief, high intensity exercise alters serum ghrelin and growth hormone concentrations but not IGF-I, IGF-II or IGF-I bioactivity. Growth Horm. IGF Res..

[120] Kelly P.J., Guelfi K.J., Wallman K.E., Fairchild T.J. (2012). Mild dehydration does not reduce postexercise appetite or energy intake. Med. Sci. Sports Exerc..

[121] Halliday T.M., White M.H., Hild A.K., Conroy M.B., Melanson E.L., Cornier M.A. (2021). Appetite and Energy Intake Regulation in Response to Acute Exercise. Med. Sci. Sports Exerc..

[122] Jürimäe J., Jürimäe T., Purge P. (2007). Plasma ghrelin is altered after maximal exercise in elite male rowers. Exp. Biol. Med..

[123] Burns S.F., Broom D.R., Miyashita M., Mundy C., Stensel D.J. (2007). A single session of treadmill running has no effect on plasma total ghrelin concentrations. J. Sports Sci..

[124] Martins C., Morgan L.M., Bloom S.R., Robertson M.D. (2007). Effects of exercise on gut peptides, energy intake and appetite. J. Endocrinol..

[125] Sartorio A., Morpurgo P., Cappiello V., Agosti F., Marazzi N., Giordani C., Rigamonti A.E., Muller E.E., Spada A. (2008). Exercise-induced effects on growth hormone levels are associated with ghrelin changes only in presence of prolonged exercise bouts in male athletes. J. Sports Med. Phys. Fit..

[126] Hagobian T.A., Sharoff C.G., Stephens B.R., Wade G.N., Silva J.E., Chipkin S.R., Braun B. (2009). Effects of exercise on energy-regulating hormones and appetite in men and women. Am. J. Physiol. Regul. Integr. Comp. Physiol..

[127] Shiiya T., Ueno H., Toshinai K., Kawagoe T., Naito S., Tobina T., Nishida Y., Shindo M., Kangawa K., Tanaka H. (2011). Significant lowering of plasma ghrelin but not des-acyl ghrelin in response to acute exercise in men. Endocr. J..

[128] Plinta R., Olszanecka-Glinianowicz M., Drosdzol-Cop A., Chudek J., Skrzypulec-Plinta V. (2012). The effect of three-month pre-season preparatory period and short-term exercise on plasma leptin, adiponectin, visfatin, and ghrelin levels in young female handball and basketball players. J. Endocrinol. Investig..

[129] Ballard T.P., Melby C.L., Camus H., Cianciulli M., Pitts J., Schmidt S., Hickey M.S. (2009). Effect of resistance exercise, with or without carbohydrate supplementation, on plasma ghrelin concentrations and postexercise hunger and food intake. Metabolism.

[130] Ghanbari-Niaki A. (2006). Ghrelin and glucoregulatory hormone responses to a single circuit resistance exercise in male college students. Clin. Biochem..

[131] Christ E.R., Zehnder M., Boesch C., Trepp R., Mullis P.E., Diem P., Décombaz J. (2006). The effect of increased lipid intake on hormonal responses during aerobic exercise in endurance-trained men. Eur. J. Endocrinol..

[132] Rämson R., Jürimäe J., Jürimäe T., Mäestu J. (2008). The influence of increased training volume on cytokines and ghrelin concentration in college level male rowers. Eur. J. Appl. Physiol..

[133] Hedayati M., Saghebjoo M., Ghanbari-Niaki A. (2012). Effects of circuit resistance training intensity on the plasma ghrelin to obestatin ratios in healthy young women. Int. J. Endocrinol. Metab..

[134] Rosenkilde M., Morville T., Andersen P.R., Kjær K., Rasmusen H., Holst J.J., Dela F., Westerterp K., Sjödin A., Helge J.W. (2015). Inability to match energy intake with energy expenditure at sustained near-maximal rates of energy expenditure in older men during a 14-d cycling expedition. Am. J. Clin. Nutr..

[135] Rämson R., Jürimäe J., Jürimäe T., Mäestu J. (2012). The effect of 4-week training period on plasma neuropeptide Y, leptin and ghrelin responses in male rowers. Eur. J. Appl. Physiol..

[136] Azizi M. (2012). Serum leptin and ghrelin changes-induced aerobic training in healthy young females. Int. J. Collab. Res. Intern. Med. Public Health.

[137] Martins C., Kulseng B., King N.A., Holst J.J., Blundell J.E. (2010). The effects of exercise-induced weight loss on appetite-related peptides and motivation to eat. J. Clin. Endocrinol. Metab..

[138] Kadoglou N.P., Fotiadis G., Kapelouzou A., Kostakis A., Liapis C.D., Vrabas I.S. (2013). The differential anti-inflammatory effects of exercise modalities and their association with early carotid atherosclerosis progression in patients with type 2 diabetes. Diabet. Med..

[139] Rosenkilde M., Reichkendler M.H., Auerbach P., Toräng S., Gram A.S., Ploug T., Holst J.J., Sjödin A., Stallknecht B. (2013). Appetite regulation in overweight, sedentary men after different amounts of endurance exercise: A randomized controlled trial. J. Appl. Physiol..

[140] Gibbons C., Blundell J.E., Caudwell P., Webb D.L., Hellström P.M., Näslund E., Finlayson G. (2017). The Role of Episodic Postprandial Peptides in Exercise-Induced Compensatory Eating. J. Clin. Endocrinol. Metab..

[141] Elerian A.E., Abdeen H., Elmakaky A., Mostafa M.S. (2020). Efficacy of gender, anaerobic exercise and low-calorie diet on leptin, ghrelin hormones and hunger perception: A comparative study. Obes. Med..

[142] Leidy H.J., Gardner J.K., Frye B.R., Snook M.L., Schuchert M.K., Richard E.L., Williams N.I. (2004). Circulating ghrelin is sensitive to changes in body weight during a diet and exercise program in normal-weight young women. J. Clin. Endocrinol. Metab..

[143] Foster-Schubert K.E., McTiernan A., Frayo R.S., Schwartz R.S., Rajan K.B., Yasui Y., Tworoger S.S., Cummings D.E. (2005). Human plasma ghrelin levels increase during a one-year exercise program. J. Clin. Endocrinol. Metab..

[144] Mizia-Stec K., Zahorska-Markiewicz B., Olszanecka-Glinianowicz M., Janowska J., Mucha Z., Holecki M., Gasiora Z. (2008). Ghrelin as a potential blood pressure reducing factor in obese women during weight loss treatment. Endokrynol. Pol..

[145] Kelishadi R., Hashemipour M., Mohammadifard N., Alikhassy H., Adeli K. (2008). Short- and long-term relationships of serum ghrelin with changes in body composition and the metabolic syndrome in prepubescent obese children following two different weight loss programmes. Clin. Endocrinol..

[146] Konopko-Zubrzycka M., Baniukiewicz A., Wróblewski E., Kowalska I., Zarzycki W., Górska M., Dabrowski A. (2009). The effect of intragastric balloon on plasma ghrelin, leptin, and adiponectin levels in patients with morbid obesity. J. Clin. Endocrinol. Metab..

[147] Gueugnon C., Mougin F., Nguyen N.U., Bouhaddi M., Nicolet-Guénat M., Dumoulin G. (2012). Ghrelin and PYY levels in adolescents with severe obesity: Effects of weight loss induced by long-term exercise training and modified food habits. Eur. J. Appl. Physiol..

[148] Kadoglou N.P., Vrabas I.S., Kapelouzou A., Lampropoulos S., Sailer N., Kostakis A., Liapis C.D., Angelopoulou N. (2012). The impact of aerobic exercise training on novel adipokines, apelin and ghrelin, in patients with type 2 diabetes. Med. Sci. Monit..

[149] Markofski M.M., Carrillo A.E., Timmerman K.L., Jennings K., Coen P.M., Pence B.D., Flynn M.G. (2014). Exercise training modifies ghrelin and adiponectin concentrations and is related to inflammation in older adults. J. Gerontol. Ser. A.

[150] Kim H.H., Kim Y.J., Lee S.Y., Jeong D.W., Lee J.G., Yi Y.H., Cho Y.H., Choi E.J., Kim H.J. (2014). Interactive effects of an isocaloric high-protein diet and resistance exercise on body composition, ghrelin, and metabolic and hormonal parameters in untrained young men: A randomized clinical trial. J. Diabetes Investig..

[151] Kang S.J., Kim J.H., Gang Z., Yook Y.S., Yoon J.R., Ha G.C., Ko K.J. (2018). Effects of 12-week circuit exercise program on obesity index, appetite regulating hormones, and insulin resistance in middleaged obese females. J. Phys. Ther. Sci..

[152] Ataeinosrat A., Haghighi M.M., Abednatanzi H., Soltani M., Ghanbari-Niaki A., Nouri-Habashi A., Amani-Shalamzari S., Mossayebi A., Khademosharie M., Johnson K.E. (2022). Effects of Three Different Modes of Resistance Training on Appetite Hormones in Males with Obesity. Front. Physiol..

[153] Campos R.M., de Mello M.T., Tock L., Silva P.L., Masquio D.C., de Piano A., Sanches P.L., Carnier J., Corgosinho F.C., Foschini D. (2014). Aerobic plus resistance training improves bone metabolism and inflammation in adolescents who are obese. J. Strength Cond. Res..

[154] Mason C., Xiao L., Imayama I., Duggan C.R., Campbell K.L., Kong A., Wang C.Y., Alfano C.M., Blackburn G.L., Foster-Schubert K.E. (2015). The effects of separate and combined dietary weight loss and exercise on fasting ghrelin concentrations in overweight and obese women: A randomized controlled trial. Clin. Endocrinol..

[155] King J.A., Wasse L.K., Stensel D.J., Nimmo M.A. (2013). Exercise and ghrelin. A narrative overview of research. Appetite.

[156] Mastorakos G., Pavlatou M., Diamanti-Kandarakis E., Chrousos G.P. (2005). Exercise and the stress system. Hormones.

[157] Broom D.R., Miyashita M., Wasse L.K., Pulsford R., King J.A., Thackray A.E., Stensel D.J. (2017). Acute effect of exercise intensity and duration on acylated ghrelin and hunger in men. J. Endocrinol..

[158] Weigle D.S., Cummings D.E., Newby P.D., Breen P.A., Frayo R.S., Matthys C.C., Callahan H.S., Purnell J.Q. (2003). Roles of leptin and ghrelin in the loss of body weight caused by a low fat, high carbohydrate diet. J. Clin. Endocrinol. Metab..

[159] Klok M.D., Jakobsdottir S., Drent M.L. (2007). The role of leptin and ghrelin in the regulation of food intake and body weight in humans: A review. Obes. Rev..

[160] Marzullo P., Verti B., Savia G., Walker G.E., Guzzaloni G., Tagliaferri M., Di Blasio A., Liuzzi A. (2004). The relationship between active ghrelin levels and human obesity involves alterations in resting energy expenditure. J. Clin. Endocrinol. Metab..

[161] Broom D.R., Batterham R.L., King J.A., Stensel D.J. (2009). Influence of resistance and aerobic exercise on hunger, circulating levels of acylated ghrelin, and peptide YY in healthy males. Am. J. Physiol. Regul. Integr. Comp. Physiol..

[162] Anderson K.C., Zieff G., Paterson C., Stoner L., Weltman A., Allen J.D. (2021). The effect of acute exercise on pre-prandial ghrelin levels in healthy adults: A systematic review and meta-analysis. Peptides.

[163] Vatansever-Ozen S., Tiryaki-Sonmez G., Bugdayci G., Ozen G. (2011). The effects of exercise on food intake and hunger: Relationship with acylated ghrelin and leptin. J. Sports Sci. Med..

[164] Douglas J.A., Deighton K., Atkinson J.M., Sari-Sarraf V., Stensel D.J., Atkinson G. (2016). Acute exercise and appetite-regulating hormones in overweight and obese individuals: A meta-analysis. J. Obes..

[165] Stensel D. (2010). Exercise, appetite and appetite-regulating hormones: Implications for food intake and weight control. Ann. Nutr. Metab..

[166] Martins C., Morgan L., Truby H. (2008). A review of the effects of exercise on appetite regulation: An obesity perspective. Int. J. Obes..

[167] Schubert M.M., Sabapathy S., Leveritt M., Desbrow B. (2014). Acute exercise and hormones related to appetite regulation: A meta-analysis. Sports Med..

[168] Hazell T.J., Islam H., Townsend L.K., Schmale M.S., Copeland J.L. (2016). Effects of exercise intensity on plasma concentrations of appetite-regulating hormones: Potential mechanisms. Appetite.

[169] Broom D.R., Stensel D.J., Bishop N.C., Burns S.F., Miyashita M. (2007). Exercise-induced suppression of acylated ghrelin in humans. J. Appl. Physiol..

[170] Leidy H.J., Dougherty K.A., Frye B.R., Duke K.M., Williams N.I. (2007). Twenty-four-hour ghrelin is elevated after calorie restriction and exercise training in non-obese women. Obesity.

[171] Stojiljkovic-Drobnjak S., Fischer S., Arnold M., Langhans W., Ehlert U. (2019). Menopause is associated with decreased postprandial ghrelin, whereas a history of anorexia nervosa is associated with increased total ghrelin. J. Neuroendocrinol..

[172] Rosenbaum M., Goldsmith R., Bloomfield D., Magnano A., Weimer L., Heymsfield S., Gallagher D., Mayer L., Murphy E., Leibel R.L. (2005). Low-dose leptin reverses skeletal muscle, autonomic, and neuroendocrine adaptations to maintenance of reduced weight. J. Clin. Investig..

[173] Blom W.A., Lluch A., Stafleu A., Vinoy S., Holst J.J., Schaafsma G., Hendriks H.F. (2006). Effect of a high-protein breakfast on the postprandial ghrelin response. Am. J. Clin. Nutr..

[174] Dhurandhar E.J., Allison D.B., van Groen T., Kadish I. (2013). Hunger in the absence of caloric restriction improves cognition and attenuates Alzheimer’s disease pathology in a mouse model. PLoS ONE.

[175] Di Girolamo F.G., Biasinutto C., Mangogna A., Fiotti N., Vinci P., Pisot R., Mearelli F., Simunic B., Roni C., Biolo G. (2024). Metabolic consequences of anabolic steroids, insulin, and growth hormone abuse in recreational bodybuilders: Implications for the World Anti-Doping Agency passport. Sports Med. Open.

[176] Lewiński A., Karbownik-Lewińska M., Wieczorek-Szukała K., Stasiak M., Stawerska R. (2021). Contribution of ghrelin to the pathogenesis of growth hormone deficiency. Int. J. Mol. Sci..

[177] Holt R.I., Sönksen P.H. (2008). Growth hormone, IGF-I and insulin and their abuse in sport. Br. J. Pharmacol..

[178] Briggs D.I., Lockie S.H., Benzler J., Wu Q., Stark R., Reichenbach A., Hoy A.J., Lemus M.B., Coleman H.A., Parkington H.C. (2014). Evidence that diet-induced hyperleptinemia, but not hypothalamic gliosis, causes ghrelin resistance in NPY/AgRP neurons of male mice. Endocrinology.

[179] Heidarianpour A., Shokri E., Baghian T., Shokri B. (2019). Benefits of aerobic training in girls with precocious puberty: Involvement of CRP and cortisol. J. Pediatr. Endocrinol. Metab..

[180] Wu W., Zhu L., Dou Z., Hou Q., Wang S., Yuan Z., Li B. (2024). Ghrelin in Focus: Dissecting Its Critical Roles in Gastrointestinal Pathologies and Therapies. Curr. Issues Mol. Biol..

[181] Algul S., Ilcin S., Ozcelik O. (2021). Effects of Excercise on Ghrelin. Prog. Nutr..

[182] Larsen P.S., Donges C.E., Guelfi K.J., Smith G.C., Adams D.R., Duffield R. (2017). Effects of Aerobic, Strength or Combined Exercise on Perceived Appetite and Appetite-Related Hormones in Inactive Middle-Aged Men. Int. J. Sport Nutr. Exerc. Metab..

[183] Yu A.P., Ugwu F.N., Tam B.T., Lee P.H., Lai C.W., Wong C.S.C., Lam W.W., Sheridan S., Siu P.M. (2018). One Year of Yoga Training Alters Ghrelin Axis in Centrally Obese Adults with Metabolic Syndrome. Front. Physiol..

[184] Mitoiu B.I., Nartea R., Miclaus R.S. (2024). Impact of Resistance and Endurance Training on Ghrelin and Plasma Leptin Levels in Overweight and Obese Subjects. Int. J. Mol. Sci..

[185] Müller T.D., Finan B., Bloom S.R., D’Alessio D., Drucker D.J., Flatt P.R., Fritsche A., Gribble F., Grill H.J., Habener J.F. (2019). Glucagon-like peptide 1 (GLP-1). Mol. Metab..

[186] Mehta R., Billings L.K., Liebl A., Vilsbøll T. (2022). Transitioning from basal-bolus or premix insulin therapy to a combination of basal insulin and glucagon-like peptide-1 receptor agonist in people with type 2 diabetes. Diabet. Med..

