# Supplementary material for: Systematic Review: Does Exercise Training Influence Ghrelin Levels?
Source: Int J Mol Sci. 2025 May 15;26(10):4753. doi: 10.3390/ijms26104753 (PMC12112022; doi:10.3390/ijms26104753)
Supplement: Supplementary file 1 [file ijms-26-04753-s001.zip › ijms-3561732-supplementary.pdf]

Supplementary Table: Risk of Bias (RoB 2.0)

Study	Randomization process	Deviations from intended interventions	Missing outcome data	Measurement of the outcome	Selection of the reported result	Overall risk of bias
Ahmadi et al., 2019	Low risk	Low risk	Low risk	Low risk	Some concerns	Low risk
Alyar et al., 2024	Low risk	Some concerns	Some concerns	Low risk	Low risk	Some concerns
Ataeinosrat et al., 2022	Low risk	Some concerns	Low risk	Some concerns	Low risk	Some concerns
Azizi, 2012	Some concerns	Low risk	Low risk	Low risk	Low risk	Some concerns
Ballard et al., 2009	Low risk	Low risk	Some concerns	Low risk	Some concerns	Low risk
Burns et al., 2007	High risk	Low risk	High risk	Low risk	Low risk	High risk
Campos et al., 2014	Low risk	Low risk	Low risk	Low risk	Low risk	Low risk
Cho et al., 2017	Some concerns	Low risk	Some concerns	Low risk	Low risk	Some concerns
Christ et al., 2006	Low risk	Low risk	Low risk	Low risk	Low risk	Low risk
Crabtree et al., 2015	Low risk	Some concerns	Some concerns	Low risk	Low risk	Some concerns
Dall et al., 2002	Some concerns	Low risk	Low risk	Low risk	Some concerns	Some concerns
Elerian et al., 2020	Low risk	Some concerns	Low risk	Low risk	Low risk	Some concerns
Erdmann et al., 2007	Low risk	Low risk	Some concerns	Low risk	Low risk	Low risk
Fico et al., 2020	High risk	Some concerns	High risk	Some concerns	Some concerns	High risk
Foster-Schubert et al., 2005	Low risk	Low risk	Low risk	Low risk	Low risk	Low risk
Ghanbari-Niaki, 2006	Low risk	Low risk	Low risk	Some concerns	Low risk	Low risk
Gibbons et al., 2017	Low risk	Low risk	Low risk	Low risk	Some concerns	Low risk
Gueugnon et al., 2012	Some concerns	Low risk	Some concerns	Low risk	Low risk	Some concerns
Hagobian et al., 2009	Low risk	Low risk	Low risk	Low risk	Some concerns	Low risk
Halliday et al., 2021	Low risk	Low risk	Low risk	Low risk	Low risk	Low risk
Hedayati et al., 2012	Some concerns	Low risk	Low risk	Low risk	Some concerns	Some concerns
Jürimäe et al., 2007a	Low risk	Low risk	Low risk	Low risk	Low risk	Low risk
Jürimäe et al., 2007	Low risk	Low risk	Low risk	Some concerns	Low risk	Low risk
Jürimäe et al., 2009	Low risk	Low risk	Low risk	Low risk	Some concerns	Low risk
Kadoglou et al., 2012	Low risk	Some concerns	Low risk	Low risk	Low risk	Some concerns
Kadoglou et al., 2013	Low risk	Low risk	Low risk	Some concerns	Low risk	Low risk
Kelishadi et al., 2008	Low risk	Low risk	Low risk	Low risk	Some concerns	Low risk
Kelly et al., 2012	Some concerns	Low risk	Low risk	Low risk	Low risk	Some concerns
Kim et al., 2014	Low risk	Low risk	Low risk	Low risk	Low risk	Low risk
Konopko-Zubrzycka et al., 2009	High risk	Some concerns	High risk	Some concerns	Some concerns	High risk
Kraemer et al., 2004	Low risk	Low risk	Low risk	Low risk	Some concerns	Low risk
Laursen et al., 2017	Low risk	Some concerns	Low risk	Low risk	Low risk	Some concerns
Leidy et al., 2004	Low risk	Low risk	Low risk	Low risk	Low risk	Low risk
Li et al., 2023	Low risk	Some concerns	Low risk	Low risk	Low risk	Some concerns
Liao et al., 2021	Low risk	Low risk	Low risk	Some concerns	Low risk	Low risk
Markofski et al., 2014	Low risk	Low risk	Low risk	Low risk	Low risk	Low risk
Martins et al., 2010	Low risk	Low risk	Low risk	Low risk	Some concerns	Low risk
Martins et al., 2007	Low risk	Low risk	Low risk	Low risk	Low risk	Low risk
Marzullo et al., 2008	Low risk	Low risk	Low risk	Some concerns	Some concerns	Some concerns
Mason et al., 2015	Low risk	Low risk	Low risk	Low risk	Low risk	Low risk
Mizia-Stec et al., 2008	Some concerns	Low risk	Low risk	Low risk	Low risk	Some concerns
Najafi et al., 2023	Low risk	Low risk	Low risk	Low risk	Low risk	Low risk
Ouerghi et al., 2019	Low risk	Low risk	Low risk	Low risk	Low risk	Low risk
Plinta et al., 2012	Low risk	Some concerns	Low risk	Some concerns	Low risk	Some concerns
Rämson et al., 2008	Some concerns	Low risk	Low risk	Low risk	Low risk	Some concerns
Rosenkilde et al., 2015	Low risk	Low risk	Some concerns	Low risk	Some concerns	Some concerns
Rosenkilde et al., 2013	Low risk	Low risk	Low risk	Low risk	Low risk	Low risk
Russel et al., 2009	Some concerns	Some concerns	Some concerns	Low risk	Some concerns	Some concerns
Sartorio et al., 2008	Some concerns	Some concerns	Some concerns	Low risk	Low risk	Some concerns
Schmidt et al., 2004	Some concerns	Low risk	Low risk	Low risk	Low risk	Some concerns
Shiiya et al., 2011	Low risk	Low risk	Low risk	Low risk	Low risk	Low risk
Stokes et al., 2010	Low risk	Low risk	Low risk	Low risk	Low risk	Low risk
Thomas et al., 2012	Low risk	Low risk	Low risk	Low risk	Some concerns	Low risk

Tobin et al., 2021	Low risk	Low risk	Low risk	Low risk	Low risk	Low risk
Toshinai et al., 2007	Low risk	Low risk	Low risk	Low risk	Low risk	Low risk
Tremblay et al., 2019	Low risk	Low risk	Low risk	Low risk	Low risk	Low risk
Zoladz et al., 2005	Low risk	Low risk	Low risk	Low risk	Low risk	Low risk
